# Real-Time Road Crack Detection on Smartphones Through ConvLSTM-Based Temporal Knowledge Distillation from a CNN-KAN and VMamba Dual-Path Network

**DOI:** 10.3390/s26165071

**Published:** 2026-08-10

**Authors:** Mengzhao Nie, Hua Huang, Mengxue Guo, Mingxia Dang, Ming Tang

**Affiliations:** 1 School of Civil Engineering and Smart Construction, Chang’an University, Xi’an 710061, China; 2 School of Civil & Architecture Engineering, Xi’an Technological University, Xi’an 710021, China; 3 Chengdu Construction Sixth Construction Engineering Co., Ltd., Chengdu 610032, China

**Keywords:** road crack detection, temporal knowledge distillation, smartphone deployment, Kolmogorov–Arnold networks, visual state space model

## Abstract

Road crack images captured by smartphones suffer from low resolution, uneven illumination, and complex background interference. Mobile devices also have limited resources for real-time high-accuracy segmentation. A two-stage framework combines a high-accuracy dual-path teacher model with a knowledge-distilled lightweight student model. The teacher model integrates a CNN-KAN path for local texture extraction and a VMamba path for global context modeling at linear complexity. A dedicated KAN-based fusion module learns adaptive nonlinear mappings between the two feature streams. On public crack datasets, the teacher model achieves an mIoU of 0.8087 and an mDice of 0.9028. It is then transferred to a self-constructed smartphone dataset built from continuous 30 fps video, where it reaches an mIoU of 0.7084 with strong robustness to illumination and blur. A GAN-based super-resolution strategy further improves the mIoU by 4.01%. A ConvLSTM-based knowledge distillation framework compresses the teacher into a lightweight MobileViT student model. This reduces the parameter count from 57.80 M to 1.57 M and cuts the GPU inference time from 99.56 ms to 1.46 ms, while retaining an mIoU of 0.7078. The deployed student model runs at 15 to 20 frames per second on an Android smartphone. An ablation study confirms that the ConvLSTM-based temporal distillation contributes beyond standard distillation. This framework provides a practical solution for real-time road crack monitoring on smartphones.

## 1. Introduction

With the accelerating aging of urban infrastructure, road crack detection has become a critical task for traffic safety management and pavement maintenance. Traditional inspection methods rely heavily on manual labor. Manual inspection is inefficient, costly, and difficult to standardize across large road networks. Beyond manual inspection, deep learning has advanced the broader field of structural health monitoring. Recent studies apply deep networks to acoustic emission source localization in composite slabs [1] and to vibration-based damage localization on large-scale bridges [2]. These approaches rely on acoustic and vibration sensing, which requires dedicated instrumentation on the structure. Image-based crack detection instead uses ordinary cameras, so it is better suited to low-cost and large-scale road monitoring. The widespread adoption of smartphones equipped with high-resolution cameras and GPS modules presents a compelling opportunity for crowd-sourced, low-cost pavement monitoring. However, images captured by smartphones under real-world conditions suffer from limited resolution, uneven illumination, motion blur, and complex background textures. These factors impose unique challenges that differ fundamentally from those addressed by existing laboratory-oriented detection methods.

Deep learning has driven substantial progress in automated crack detection from images. Convolutional neural networks (CNNs) have become a mainstream approach owing to their strong local feature extraction capability and hierarchical representation learning [3]. A review by Deng et al. [4] confirms that deep learning methods outperform traditional image processing techniques in pixel-level crack segmentation. The review also identifies data scarcity, complex background interference, and cross-scene generalization as key unresolved challenges. Ali et al. [5] further validate the advantage of encoder–decoder architectures such as UNet in pixel-level crack segmentation. Matarneh et al. [6] evaluate ten pre-trained CNN models and find that DenseNet201 combined with the Gray Wolf Optimizer achieves 98.73% accuracy in crack classification. This result confirms the effectiveness of transfer learning with feature selection. Liu [7] demonstrates that UNet variants can balance accuracy and computational efficiency by integrating different CNN encoders. Despite these advances, CNN architectures are inherently constrained by limited receptive fields. This constraint makes it difficult to capture the long-range structural continuity of real road crack patterns.

Transformer-based architectures have addressed this limitation through global self-attention mechanisms. Guo et al. [8] propose a Swin Transformer and UperNet fusion model that significantly improves detection of long and small cracks. Wang et al. [9] develop a weakly supervised hybrid network with multi-attention mechanisms to enhance both local and global feature extraction for pavement cracks. Wang [10] integrates the Swin Transformer with convolutional layers in SwinCrack. The model achieves strong performance on multiple public datasets by jointly modeling global dependencies and local texture. However, the computational complexity of standard Transformers grows quadratically with input resolution. This growth makes efficient deployment on resource-constrained mobile devices difficult for the high-resolution images typically produced by smartphones.

The deployment challenge is further compounded by the scale of modern vision models. These models typically contain tens to hundreds of millions of parameters, which makes direct inference on mobile devices impractical [11]. For smartphone-based road crack detection under real-time field conditions, the gap between server-grade model performance and mobile hardware capability remains a central obstacle.

Two recent architectural advances show strong potential to address these limitations. Kolmogorov–Arnold Networks (KAN) [12] are grounded in the Kolmogorov–Arnold representation theorem. They replace fixed activation functions with learnable univariate nonlinear functions. This design achieves stronger feature representation with fewer parameters than conventional MLP-based designs. Li et al. [13] demonstrate that KAN achieves superior performance in medical image segmentation, particularly at complex boundaries. Yang et al. [14] propose SKPNet, a KAN-based network that enhances the perception of bridge cracks in semantic segmentation. The Mamba model introduced by Gu and Dao [15] resolves the quadratic complexity of Transformers by adopting a selective state space model with linear time complexity. VMamba, designed as a vision-specific backbone by Liu et al. [16], achieves global modeling capability comparable to Transformers while maintaining linear computational complexity and significantly reduced parameter counts. Han et al. [17] propose MambaCrackNet, which applies visual Mamba to pixel-level crack segmentation and improves fine crack detection. These features make KAN and VMamba well suited as building blocks of a parameter-efficient teacher model for smartphone-based crack detection. However, existing KAN- and Mamba-based crack segmentation methods adopt single-path designs oriented toward server-side inference. The joint fusion of the two architectures has not been established. The compression required for smartphone deployment has not been addressed. The temporal continuity available in smartphone video streams also remains unexploited.

Nevertheless, KAN and VMamba each have complementary weaknesses when processing low-quality smartphone images independently. KAN is sensitive to high-frequency noise and non-uniform illumination [18], while Mamba-based models show limited precision for slender and elongated crack structures. These limitations motivate a dual-path design in which both architectures reinforce each other through a dedicated fusion mechanism.

To address these challenges, this study proposes a two-stage framework for real-time road crack detection on smartphones. The framework couples a high-accuracy teacher model with a lightweight deployable student model, so that segmentation accuracy and mobile efficiency are achieved together. In the first stage, a dual-path encoder–decoder network is constructed as a high-accuracy teacher model. It combines a CNN-KAN path for local texture extraction with a VMamba path for global context modeling. The two paths are connected through a KAN-based fusion module, which learns adaptive nonlinear mappings between the heterogeneous feature streams. This joint design fills the gap left by earlier single-path KAN and Mamba models. Since smartphone-based acquisition naturally produces continuous video streams rather than isolated images, the neighboring frames provide an additional source of supervision that conventional distillation frameworks do not exploit. In the second stage, a ConvLSTM-based structured knowledge distillation framework compresses the teacher model into a mobile-deployable student model. This reduces the parameter count from 57.80 M to 1.57 M and the inference time from 99.56 ms to 1.46 ms while preserving detection accuracy. This step bridges the common separation between accuracy-oriented models and deployment-oriented models. The influence of three GAN-based enhancement strategies on real-world smartphone imagery is further investigated, and super-resolution reconstruction is identified as the most effective strategy. This framework provides a practical and scalable approach for smartphone-assisted intelligent transportation infrastructure monitoring.

## 2. Methods and Datasets

**Figure 1:**
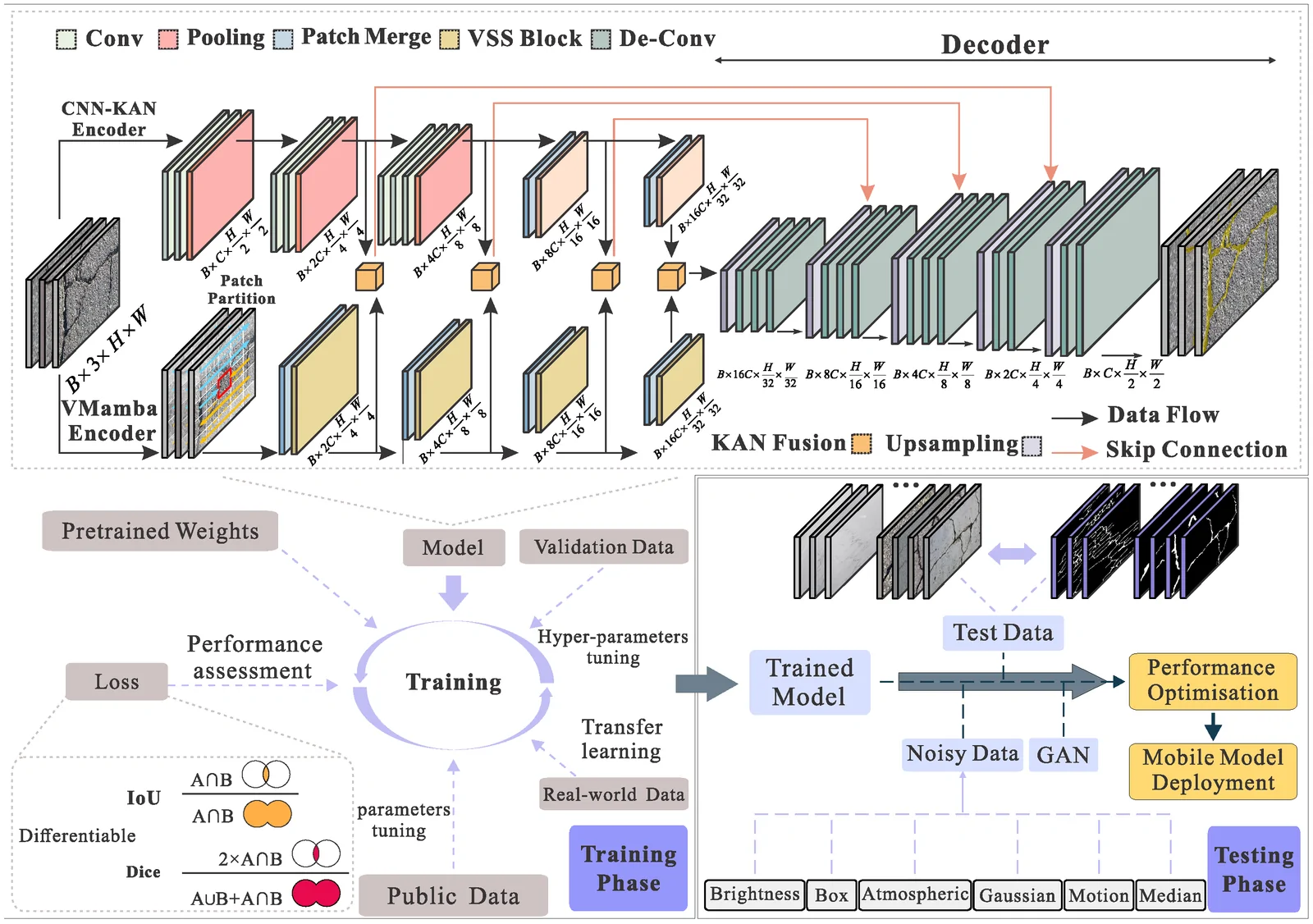
Overall framework of the proposed two-stage crack detection system.

This section details the architecture of the proposed teacher model and the datasets used for training and evaluation. The model adopts a dual-path encoder–decoder structure combining a CNN-KAN path and a VMamba path. The two paths are connected through a KAN-based fusion module to form a complete segmentation framework. Figure 1 illustrates the overall technical route of this study.

### 2.1. Model Feature Extraction

#### 2.1.1. Kolmogorov–Arnold Enhanced Convolutional Neural Network

**Figure 2:**
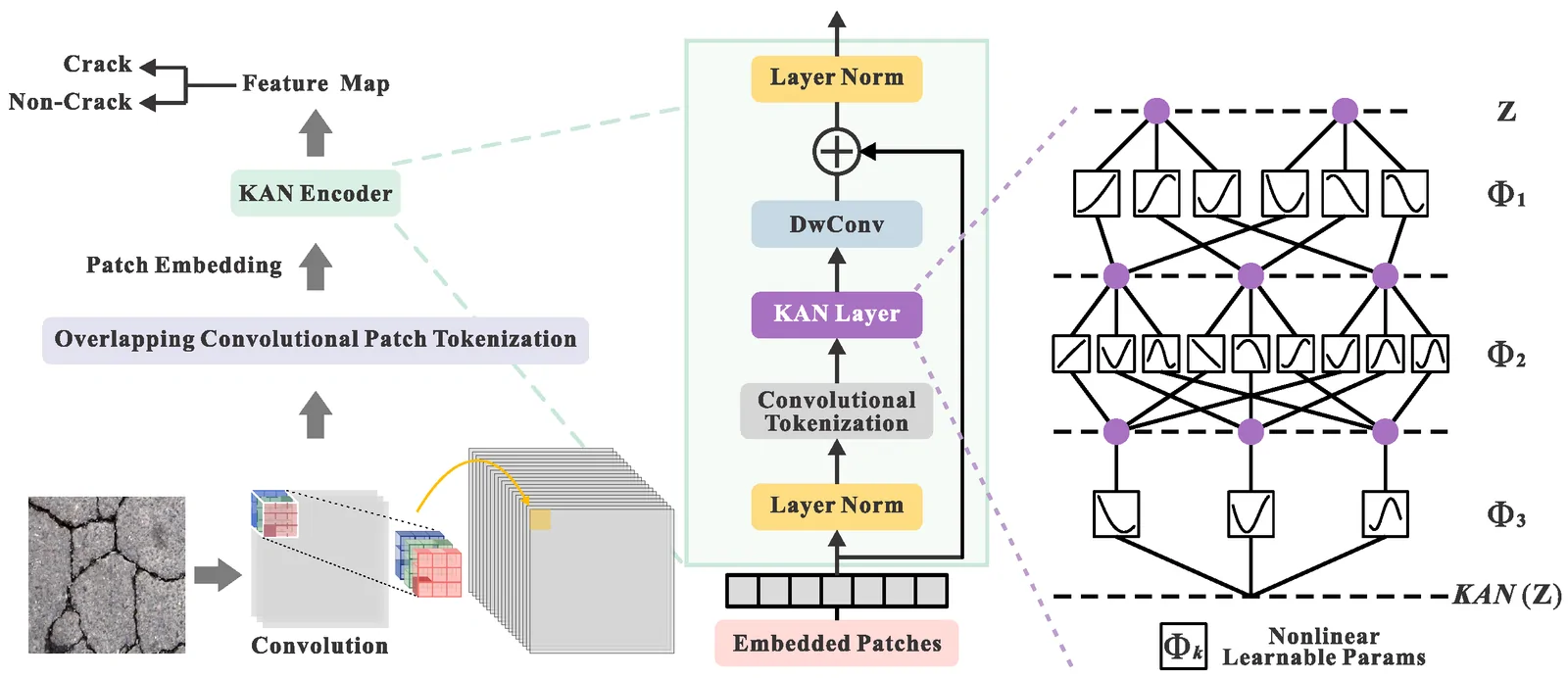
Overview of the integrated CNN-KAN architecture for feature encoding.

The CNN-KAN path is constructed by embedding KAN layers into the convolutional feature extraction pipeline. This replaces conventional fixed activation functions, such as ReLU and Sigmoid, with learnable univariate nonlinear functions grounded in the Kolmogorov–Arnold representation theorem. This design enables each connection to adaptively learn more complex feature transformations without increasing the number of convolutional filters. The result is richer nonlinear modeling capability at a comparable parameter cost. Standard CNN architectures face particular difficulty when processing smartphone-captured crack images. In such images, low resolution, varying illumination, and complex background textures demand strong nonlinear representational capacity across channels. The introduction of KAN layers directly addresses this limitation by replacing rigid kernel designs with adaptive activation functions suited to the high variability of real-world pavement images. Figure 2 illustrates the feature extraction process of the CNN-KAN path.

Spatially localized features are first extracted using a standard convolutional layer. This layer captures the texture, edges, and underlying features of the image. Convolutional neural networks perform convolutional operations with a series of trainable filters. These filters capture local dependencies in the input image. The filters traverse the image and convert it into an internal feature map, as shown in (1) [19].

(1) $$O\left(i,\;j\right)=\sum_{k=1}^{m}\sum_{l=1}^{n}I\left(i+k-1,\;j+l-1\right)\;K\left(k,\;l\right),$$

where *I* represents an input image of dimensions M×N; *K* denotes a filter of size m×n; *O* signifies a feature map of dimensions (M−m+1)×(N−n+1).

Following convolutional feature extraction, tokenization is first performed by reconstructing the output features of the convolutional phase $X_{L}$ into a sequence of flat 2D patches $\left\{X_{L}^{i}\in R^{P^{2}\cdot C_{L}}i=1,\ldots N\right\}$. The size of each patch is P×P, where $N=\frac{H_{L}\times \;W_{L}}{P^{2}}$ represents the number of feature patches. The vectorized blocks are then mapped into a potential D-dimensional embedding space using a trainable linear projection $E\in R^{\left(P^{2}\cdot C_{L}\right)\times D}$ as shown in Equation (2) [20].

(2) $$Z_{0}=\left[X_{L}^{1}E;\;X_{L}^{2}E;\;\cdots ;\;X_{L}^{N}E\right],$$

The KAN layer replaces the fixed nonlinear transformations of standard MLP layers with k nested learnable univariate activation functions. These functions can be parameterized using spline interpolation or Fourier series to fit arbitrary nonlinear relationships with greater parameter efficiency. This property makes the KAN layer more suitable for lightweight feature extraction in smartphone-captured crack images [21]. The KAN layer is defined in Equation (3).

(3) $$\mathrm{KAN}(Z)=\left(\Phi_{K-1}\circ \Phi_{K-2}\circ \cdots \circ \Phi_{1}\circ \Phi_{0}\right)Z,$$

where $\Phi_{i}$ denotes the i-th layer of the entire KAN network. Each KAN network layer has $n_{in}$-dimensional inputs and $n_{out}$-dimensional outputs. Each layer $\Phi_{i}$ consists of $n_{in}\times n_{out}$ learnable univariate activation functions $\phi_{q,p}$, where $p=1,2,\cdots ,n_{in}$ and $q=1,2,\cdots ,n_{out}$.

#### 2.1.2. Vision Mamba

**Figure 3:**
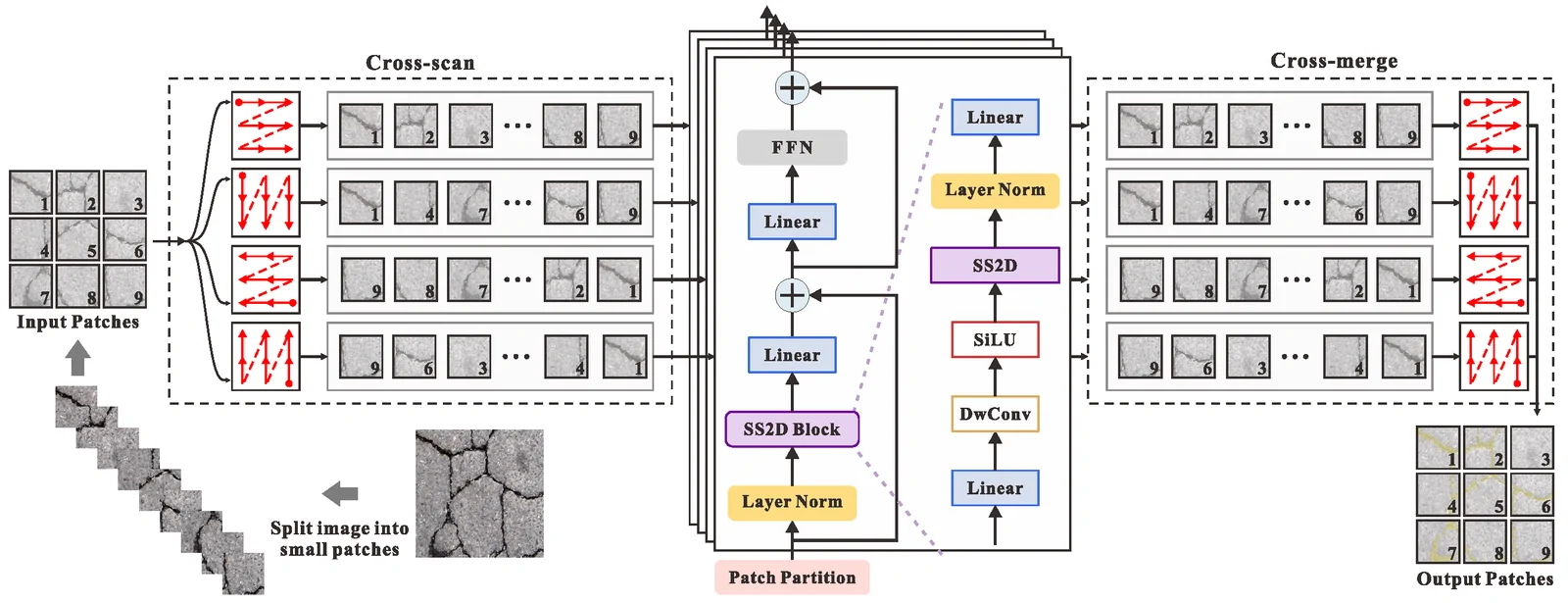
Architecture of VMamba with the 2D selective scanning module.

Standard Transformer architectures capture global dependencies through self-attention mechanisms. However, their quadratic computational complexity $O(N^{2})$ makes them unsuitable for lightweight deployment on mobile devices. VMamba resolves this by adopting a selective state space model with linear complexity O(N). This maintains global receptive field coverage at significantly lower computational cost. To handle the inherently two-dimensional nature of visual data, VMamba employs a two-dimensional selective scanning module (SS2D) [22]. This module traverses input patches along four complementary directions: horizontal forward, horizontal reverse, vertical forward, and vertical reverse. This ensures omnidirectional context capture. Figure 3 illustrates this scanning process.

The overall architecture of VMamba follows a hierarchical design in which multiple Visual State Space (VSS) blocks serve as the basic computational units [23], as described in Equation (4).

(4) $$Z_{k}=\mathrm{LN}\left(Z_{k-1}+\mathrm{SS}2D\left(Z_{k-1}\right)\right),$$

where LN denotes layer normalization and SS2D is the 2D selective scanning operation described above. In practice, the VSS block may also contain feed-forward networks and depth-wise convolutions to enhance local feature extraction. For the input feature map $Z\in R^{H\times W\times C}$, SS2D expands the feature map along four complementary directions: horizontal forward, horizontal reverse, vertical forward, and vertical reverse. This ensures omnidirectional context capture for each pixel. The selective state-space module is then applied independently to each sequence. Historical information is recursively propagated through the hidden state h(t), forming an effective global receptive field, as described in Equation (5) [17].

(5) $$h^{\prime }\left(t\right)=\mathrm{Ah}\left(t\right)+Bu\left(t\right),\;y\left(t\right)=\mathrm{Ch}\left(t\right)+Du\left(t\right),$$

where A represents the state transition matrix, B is the input mapping matrix, C denotes the output mapping matrix, and *D* is the skip connection parameter. In practice, the continuous system in Equation (5) is discretized through the zero-order hold method with a learnable step size Δ, yielding the discrete parameters $\bar{A}$ and $\bar{B}$. The selective mechanism further makes B, C, and Δ functions of the input, which allows the model to selectively retain or discard information along each scanning path. Unlike self-attention, VMamba conveys contextual information through the compressed hidden state rather than computing attention scores among all tokens. This reduces the complexity from $O(N^{2})$ to O(N) and lowers the computational burden on mobile hardware. The four processed sequences are then fused and reconstructed into a 2D feature map that maintains complete spatial structure.

#### 2.1.3. KAN Fusion Module

The CNN-KAN encoder and the VMamba encoder produce heterogeneous feature representations that differ significantly in inductive bias and information encoding. Simple concatenation or linear fusion is therefore insufficient to fully exploit their complementary advantages. To address this, a dedicated KAN-based fusion module is introduced to learn adaptive nonlinear mappings between the two feature streams [24]. The features extracted by the CNN-KAN encoder ($Z_{1}\in R^{H_{i}\times W_{i}\times C_{1}^{i}}$) and those extracted by VMamba ($Z_{2}\in R^{H_{i}\times W_{i}\times C_{2}^{i}}$) are normalized and reshaped into a combined representation $Z\in R^{H_{i}\times W_{i}\times \left(C_{1}^{i}+C_{2}^{i}\right)}$. This combined representation serves as input to a multilayer KAN transformation [25], as formulated in Equation (6):

(6) $$Z_{\mathrm{fused}}={\mathrm{KAN}}_{\mathrm{fuse}}\left(\left[\mathrm{LN}\left(Z_{1}\right);\;\mathrm{LN}\left(Z_{2}\right)\right]\right),$$

where LN denotes layer normalization and ${\mathrm{KAN}}_{\mathrm{fuse}}$ denotes the multilayer KAN transformation of the fusion module. The concatenated result corresponds to the combined representation Z defined above.

**Figure 4:**
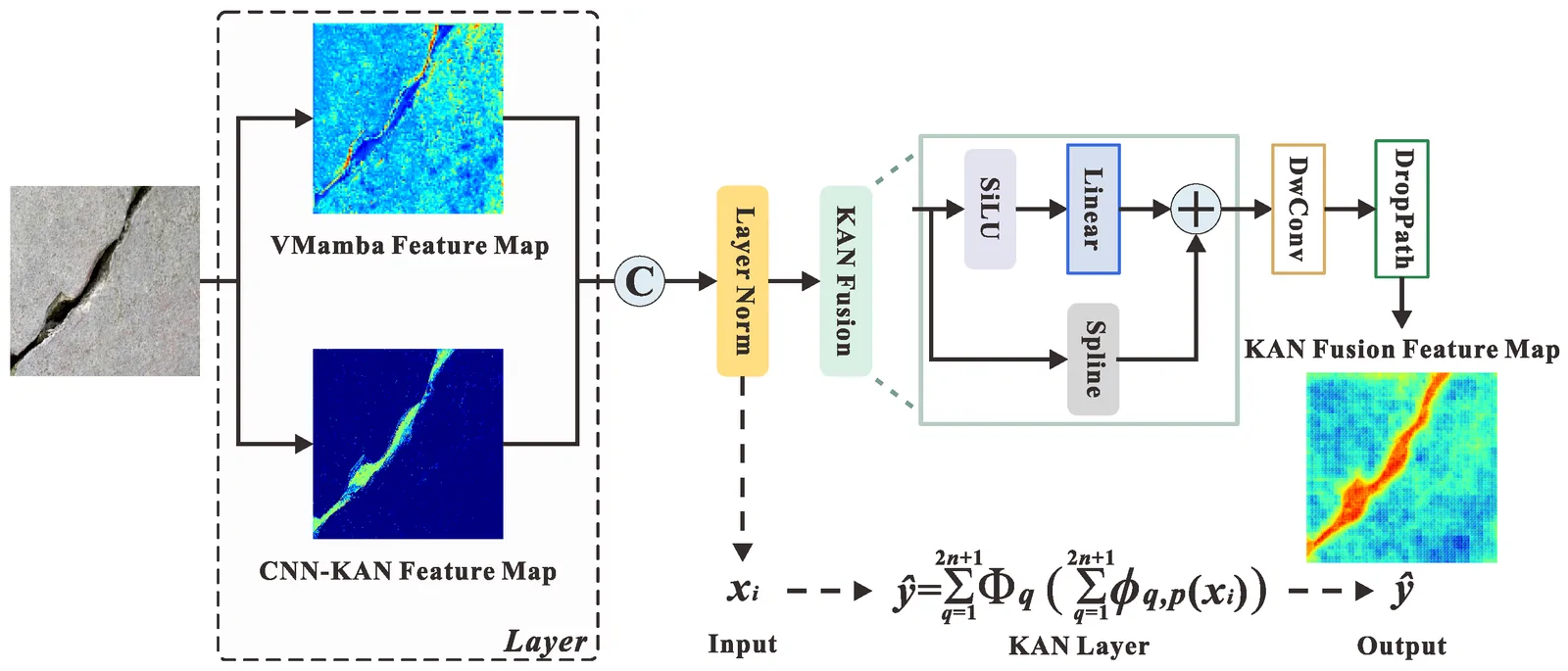
Structure of the KAN-based fusion module.

Unlike linear fusion, the KAN transformation applies learnable nonlinear mappings across *k* layers. It selectively emphasizes informative features while suppressing redundancy, as illustrated in Figure 4. This produces a fused representation that combines local texture detail from the CNN-KAN path with global contextual information from the VMamba path, retaining the complementary advantages of both encoders.

### 2.2. KAN Fusion-Based Dual-Path Neural Network for Crack Detection

**Figure 5:**
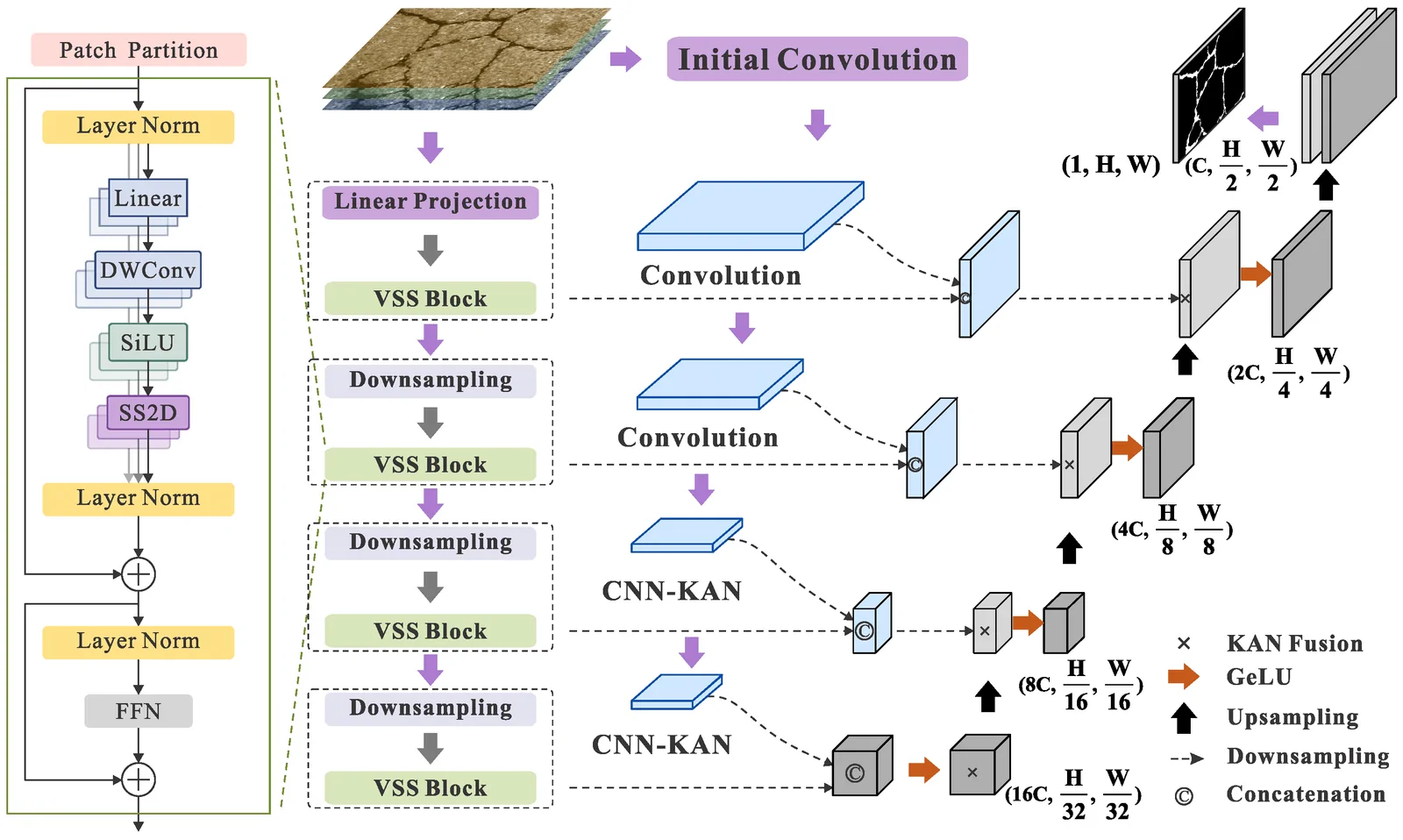
Overview of the dual-path encoder–decoder architecture with the KAN-based fusion module.

Figure 5 illustrates the proposed dual-path encoder–decoder architecture, which constitutes the teacher model for smartphone-based crack detection. The encoder adopts a hierarchical feature extraction strategy combining the CNN-KAN path and the VMamba path. At shallow layers, the CNN-KAN path extracts local texture and fine-grained crack details from smartphone-captured images. At deeper layers, the VMamba path performs multi-scale feature extraction through alternating linear projection and VSS blocks, modeling global structural context at linear computational cost. The KAN-based fusion module then integrates the complementary representations from both paths at each encoder stage. The dual-path design therefore refers to the two parallel encoder branches, whereas the KAN-based fusion module is the distinct component that combines their features.

The decoder progressively restores feature maps from (16C,H/32,W/32) to the final segmentation mask (1,H,W) through UNet-style skip connections at each upsampling stage. These connections adaptively balance high-level semantic information with low-level spatial detail. This balance ensures accurate recovery of the fine and irregular crack morphologies in smartphone-captured pavement images. The resulting segmentation output of this teacher model subsequently guides the knowledge distillation process into the mobile-deployable student model [26].

### 2.3. Datasets Description

The proposed framework is evaluated under two data settings. The first setting combines four publicly available benchmark datasets, namely Crack500, DeepCrack, CFD, and CrackTree [27,28,29,30], with accurate pixel-level annotations and diverse crack morphology. Since the original CrackTree annotations are thin centerline curves, the crack masks of this dataset are re-delineated into region-level masks based on the original skeletons to be consistent with the region annotations of the other three datasets. All images are resized to a uniform resolution of 448×448, and the combined set contains 12,887 images. The combined public dataset is randomly divided into a training subset of 9021 images, a validation subset of 1933 images, and a test subset of 1933 images at a ratio of 70% to 15% to 15%. The validation subset is used for the dynamic weighting in Equation (7) and for model selection, and it is never used for parameter updates. These data are used to train and evaluate the teacher model and to conduct ablation experiments analyzing the contribution of each architectural component.

**Figure 6:**
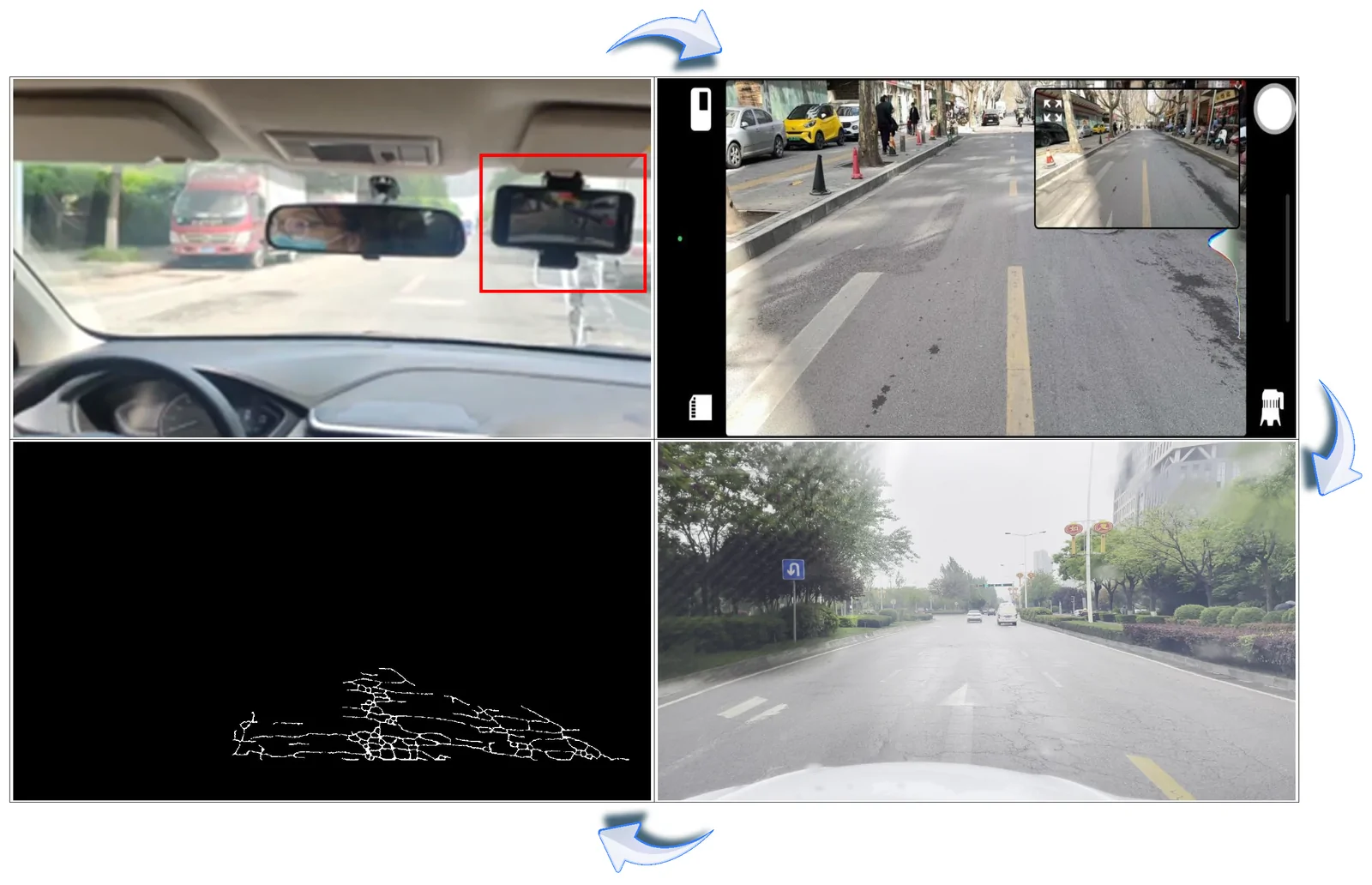
Smartphone-based data collection and processing.

The second setting is a self-constructed asphalt pavement dataset collected in Xi’an, Shaanxi Province, China, to evaluate performance under real-world smartphone imaging conditions, as shown in Figure 6. The raw data are recorded as continuous video streams at a rate of 30 frames per second and a resolution of 1920×1080. The videos were recorded with an iPhone 14 Pro Max, fixed to the windshield of a passenger car with a suction mount. The vertical distance between the camera and the road surface was approximately 1.5 m, and the camera was oriented at a pitch angle of approximately 25 degrees relative to the road surface. The vehicle speed was kept at about 30 km/h during acquisition. The pavement sections consist mainly of aged asphalt surfaces with visible wear, and a small number of newly paved sections are also included. Guided by the GPS coordinates recorded during driving, 1769 keyframes are extracted from the video streams along the driving route. Because the camera looks ahead along the road, adjacent keyframes partially overlap, and the same crack may appear in two neighboring keyframes. The annotation protocol was designed to improve label reliability through independent cross-checking. Each keyframe was first labeled by one of five trained annotators, and two additional annotators then independently reviewed the label. A keyframe was accepted only when both reviewers agreed with the initial annotation, and any disagreement triggered group discussion and re-annotation until the whole team reached consensus. This three-stage procedure was applied to all 1769 keyframes. The dataset provides pixel-level binary masks of crack regions rather than crack-type labels. The study targets binary crack segmentation, so a type label is not required by the segmentation objective or by the loss functions. A crack-type annotation layer is left for future work. The annotated frames cover diverse crack morphologies under varying illumination, viewing angles, and background conditions that reflect the challenges of practical smartphone-based detection. For the ConvLSTM-based temporal distillation, short clips of frames sampled around each annotated keyframe are additionally used. Only the keyframe in each clip carries pixel-level annotation. The construction of these clips and their role in the distillation are described in Section 3.2.

The smartphone dataset is divided into a training subset of 1239 keyframes, a validation subset of 265 keyframes, and a test subset of 265 keyframes at a ratio of 70% to 15% to 15%. The split is performed by road segment rather than by individual frame. All keyframes from the same road segment are assigned to the same subset, so overlapping keyframes never cross subset boundaries. This grouping prevents the leakage of near-duplicate frames between training and testing. The unannotated neighboring frames used for temporal distillation inherit the subset assignment of their corresponding keyframe. No frame associated with a test location is exposed during training. All smartphone-domain results in the following sections, including the baseline comparisons, the ablation studies, and the student model evaluations, are reported on this fixed test subset. This dataset serves as the primary benchmark for evaluating the teacher model under transfer learning and for assessing the deployed student model under mobile hardware constraints.

## 3. Model Implementation and Optimization Evaluation

### 3.1. Optimization of Generative Adversarial Networks

**Figure 7:**
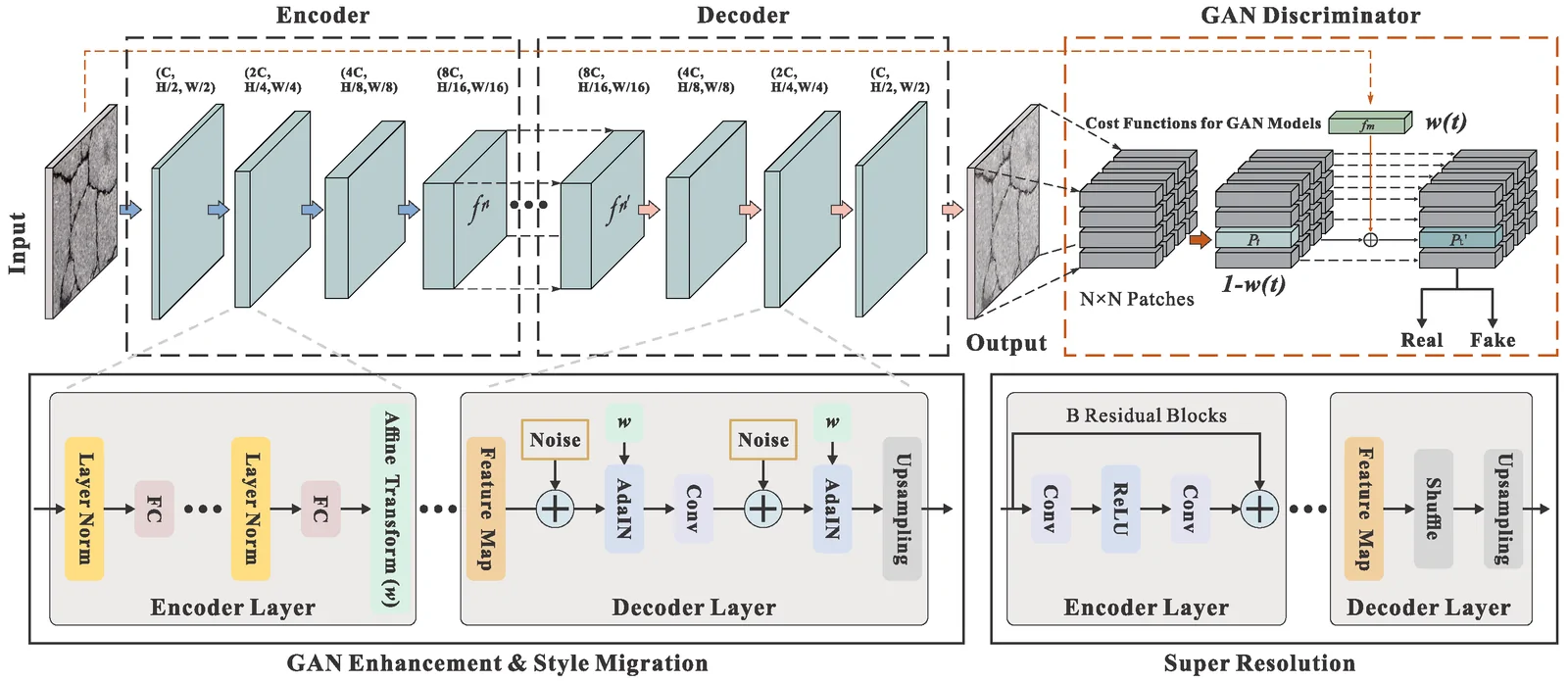
Optimization framework for pavement crack segmentation with generative adversarial networks.

To investigate the influence of GAN-based data augmentation on real-world pavement imagery, three complementary enhancement strategies are examined for the teacher model, as shown in Figure 7. All three strategies are applied as training-time augmentation. No GAN-based processing is applied to the input images during inference. A dynamic weighting strategy balances the contribution of original and GAN-generated data throughout training by adjusting their ratio based on model sensitivity, as formulated in Equation (7).

(7) $$w\left(t\right)=w_{0}+\Delta w\cdot \left(\mathrm{sigmoid}\left(S_{\mathrm{orig}\;}\left(t\right)-S_{\mathrm{gan}\;}\left(t\right)\right)-0.5\right),$$

where $w_{0}$ is the base weight value of 0.5 and Δw denotes the maximum weight adjustment. The value of Δw is set to 1. This setting constrains w(t) within the range of 0 to 1. These two values are fixed by this range requirement rather than tuned as free hyperparameters. The weight w(t) is assigned to the original data. The complementary weight 1−w(t) is assigned to the GAN-generated data. $S_{\mathrm{orig}\;}\left(t\right)$ and $S_{\mathrm{gan}\;}\left(t\right)$ represent the sensitivity scores of the current model on the original and GAN-generated validation data, respectively. These scores are calculated from the area under the precision–recall curve to maintain high sensitivity to small cracks. Higher weights are assigned to original data in early training to establish a stable foundation. These weights are then dynamically adjusted as the model adapts to both data sources.

GAN-based image enhancement diversifies the training distribution by generating augmented crack samples that strengthen crack edge information [31]. CycleGAN-based style migration then performs bidirectional mapping between a high-quality source domain and the smartphone target domain, preserving crack geometry while adapting illumination and contrast to improve cross-device generalization [32]. Super-resolution reconstruction further addresses the low resolution of smartphone imagery by recovering high-frequency crack details through residual dense blocks and enhanced perceptual loss, enabling detection of millimeter-level fine cracks [33].

### 3.2. Model Distillation

**Figure 8:**
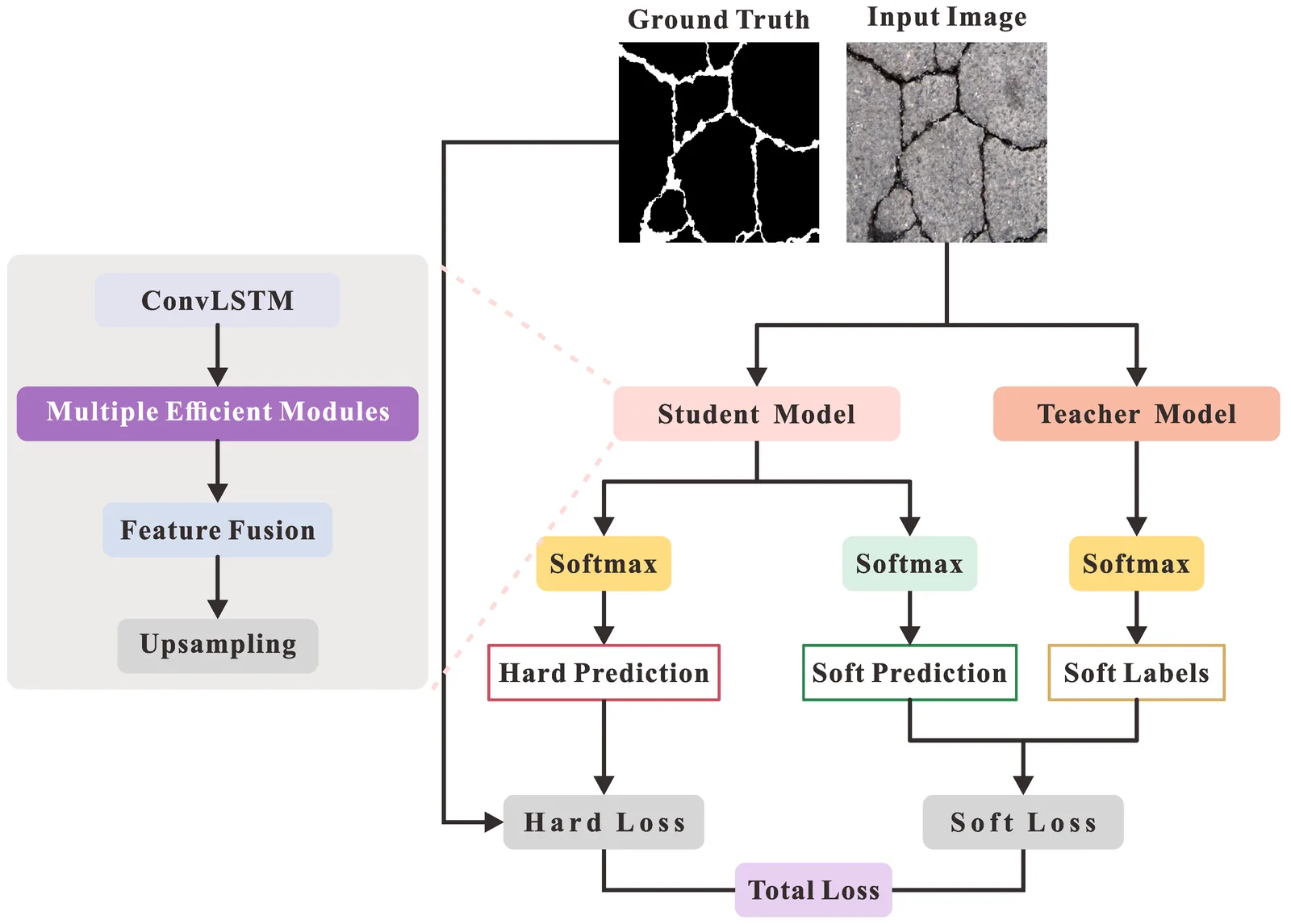
Structure of the ConvLSTM-based knowledge distillation framework.

Building upon the teacher model, a ConvLSTM-based structured knowledge distillation framework is proposed to compress the network into a mobile-deployable student model, as shown in Figure 8. Unlike conventional knowledge distillation approaches that treat each image independently, this framework is specifically designed for the spatio-temporal characteristics of smartphone video streams. ConvLSTM is incorporated to capture crack continuity and morphological changes across successive frames. Each temporal clip contains three frames, namely the annotated keyframe together with the preceding and following frames sampled along the GPS trajectory. The frames are sampled at an interval of about 4 m of vehicle travel rather than at a fixed time step. Because the vehicle moves forward, adjacent frames overlap only partially. The lower region of the keyframe overlaps the upper region of the preceding frame, and the upper region of the keyframe overlaps the lower region of the following frame. A crack is therefore not always visible in all three frames, but a crack that falls in an overlapping region is observed across adjacent frames. This partial overlap provides the temporal cues that the ConvLSTM module uses to model crack continuity. A clip length of three frames provides sufficient temporal context, while it keeps the memory and computation of the distillation stage low. Only the keyframe carries a manual pixel-level annotation, so the neighboring frames cannot be supervised by ground-truth labels. To supervise these frames, the trained teacher model is applied to every frame of the clip, including the two unlabeled neighbors. For each frame, the teacher produces a soft segmentation map and intermediate features, which serve as the distillation targets for the student. The manual annotation of the keyframe is used only for the task loss, while the teacher soft labels supervise the student on all three frames. In this way, the ConvLSTM receives supervision on the unlabeled neighbor frames from the teacher rather than from manual labels. Three key layers of the teacher model are selected for knowledge extraction. These layers are the outputs of the KAN fusion module at three depths of the encoder. They correspond to the low-level feature layer, the mid-level semantic layer, and the high-level decision layer. These three depths span the shallow, intermediate, and deep stages of the encoder. Together, they form a multi-scale knowledge transfer pipeline that ensures comprehensive transfer of crack detection capability.

The student model is first trained with the task loss alone to establish a baseline. The distillation proceeds in three stages. Feature distillation then minimizes the difference between intermediate layer features of the teacher and student models, with the DWConv module as the primary alignment target for transferring local structural crack knowledge [34]. Response distillation subsequently minimizes the output distribution divergence between the two models to replicate the teacher’s segmentation decisions. Temporal relationship distillation is then applied through the ConvLSTM module, which models the temporal relationship of crack features across the frames of a clip. This temporal stage complements the feature-level and response-level distillation that standard frameworks apply to single images. The task loss and the three distillation losses are combined into a single training objective. The task loss has a weight of 1.0. The feature, response, and temporal distillation losses each have a weight of 0.5. The response distillation uses a temperature of 1.0. The distilled student model is deployed on smartphones via the ncnn framework, achieving real-time inference while retaining detection accuracy comparable to the teacher model.

### 3.3. Segmentation Model Training and Evaluation

#### 3.3.1. Optimization and Model Initialization

The proposed framework is implemented in PyTorch [35]. The Adam optimizer is adopted with an initial learning rate of 0.001. The learning rate is adjusted dynamically through a cosine annealing strategy with warm restarts. The first restart occurs at the 15th epoch, and each subsequent restart interval doubles the previous one. Both the teacher model and the student model are trained for 100 epochs with a batch size of 8. Data augmentation is applied during training to improve generalization on smartphone-captured crack images. It is not applied during testing.

All models are trained on a workstation equipped with an NVIDIA GeForce RTX 4090 GPU with 24 GB of memory. The inference times reported in the following sections are measured on the same GPU with a batch size of 1 at an input resolution of 448×448. Each reported value is averaged over repeated inference runs after warm-up. The on-device performance of the deployed student model is measured separately on a Samsung Galaxy S21 smartphone with a Qualcomm Snapdragon 888 chip. The deployed model is built with the ncnn framework, version 1.0 of build 20241226. The deployed model processes the live camera stream of the phone in real time. Each frame is resized to the network input resolution of 448×448 before inference, which is the same input resolution as the GPU measurement. The latency is measured at room temperature under a normal steady thermal state.

#### 3.3.2. Model Evaluation Metrics

Five metrics are used to evaluate both the teacher model and the distilled student model. Precision ($P=\frac{T_{P}}{T_{P}+F_{p}}$) and recall ($R=\frac{T_{P}}{T_{P}+F_{N}}$) measure detection correctness and completeness, respectively, where $T_{P}$, $F_{P}$, and $F_{N}$ denote true positive, false positive, and false negative. These two metrics are combined into the F1 score ($F_{1}=\frac{2\times P\times R}{P+R}$). Intersection over union ($\mathrm{IoU}=\frac{T_{P}}{T_{P}+F_{P}+F_{N}}$) and the Dice coefficient ($\mathrm{Dice}=\frac{2T_{P}}{2T_{P}+F_{P}+F_{N}}$) quantify the overlap between predicted and ground truth crack regions. The mean Dice coefficient (mDice) reflects overall segmentation performance. In this study, every metric is computed on the crack class only rather than averaged over the crack and background classes. A class-averaged score would be dominated by the background, and it would hide the performance on the sparse crack pixels. The mIoU and mDice denote the mean of the crack-class IoU and Dice over all test images, and the corresponding standard deviations are reported where applicable. The reported mIoU is therefore the crack-class IoU, not a class-averaged value. The mean IoU (mIoU) penalizes segmentation errors more strictly. It serves as the primary metric for evaluating model performance under the challenging imaging conditions of smartphone-captured pavement images.

#### 3.3.3. Loss Function

A combined loss function of IoU loss and Dice loss is employed to train both the teacher model and the student model. This combination is selected for its effectiveness in handling unclear boundaries and small target regions that are characteristic of fine cracks in smartphone-captured pavement images. By jointly minimizing IoU loss and Dice loss, the model improves overlap between predicted and ground truth crack regions. This also reduces false positives and false negatives [36].

The final loss function used for the proposed network is as follows.

(8) $$\;\text{IoU\_Loss}\;=1-\frac{\sum_{i=1}^{N}p_{i}t_{i}+\varepsilon }{\sum_{i=1}^{N}p_{i}+\sum_{i=1}^{N}t_{i}-\sum_{i=1}^{N}p_{i}t_{i}+\varepsilon },$$

(9) $$\;\text{Dice\_Loss}\;=1-\frac{2\sum_{i=1}^{N}p_{i}t_{i}+\varepsilon }{\sum_{i=1}^{N}p_{i}+\sum_{i=1}^{N}t_{i}+\varepsilon },$$

(10)  Loss = Dice_Loss + IoU_Loss,

where *N* represents the number of all pixels in the batch, $p_{i}$ represents the predicted outcome of pixel *i*, $t_{i}$ represents the underlying true value of pixel *i*, and ε represents the smoothing factor. In practice, the smoothing factor is usually added to prevent the denominator from being predicted as zero. It also smooths the loss and the gradient. The value of ε is set to 1.

## 4. Experimental Results and Analysis

### 4.1. Model Evaluation on Public Datasets

The proposed method is first evaluated on four public benchmark datasets. These datasets are Crack500, DeepCrack, CFD and CrackTree. The per-dataset results are reported in Table 1. The proposed method is compared against eleven baseline models from four categories. The KAN-based model is SKPNet [14], and the Mamba-based model is MambaCrackNet [17]. The CNN-based models are UNet [37], ResUNet [38], PSPNet [39], FPN [40], DeepLabV3 [41], and DeepLabV3+ [42]. The Transformer-based models are TransUNet [43], CrackFormer [44], and DefNet [45]. The per-dataset evaluation allows the segmentation performance of each model to be assessed under different crack morphologies and imaging conditions. Real-world road cracks exhibit diverse morphologies that no single dataset can fully represent. The four public datasets are therefore combined into a unified dataset to train the teacher model. This teacher model is then transferred to the real-world smartphone-captured pavement dataset in the following sections.

**Figure 9:**
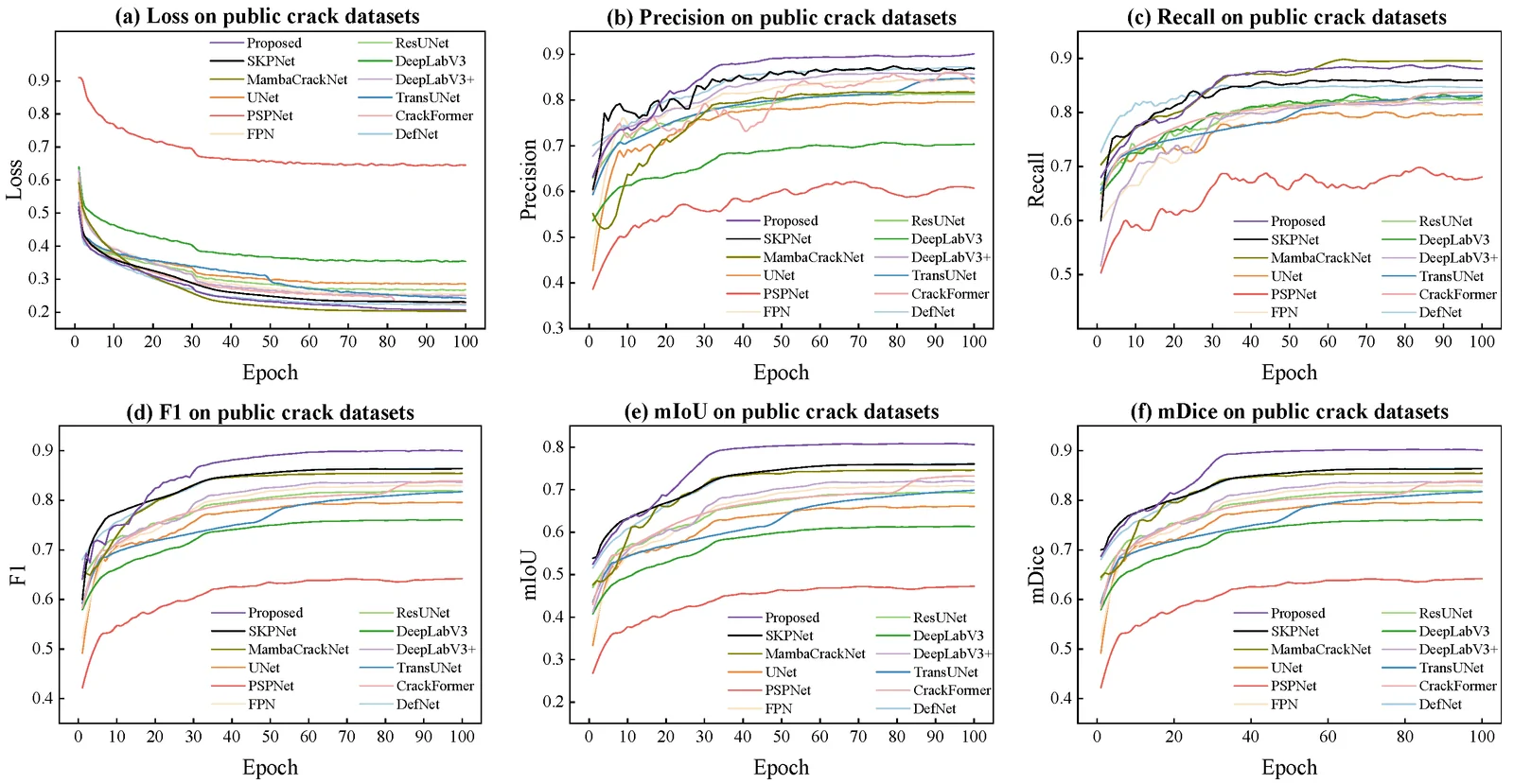
Training curves of the evaluated models on the combined public dataset: (**a**) loss; (**b**) precision; (**c**) recall; (**d**) F1; (**e**) mIoU; (**f**) mDice.

**Figure 10:**
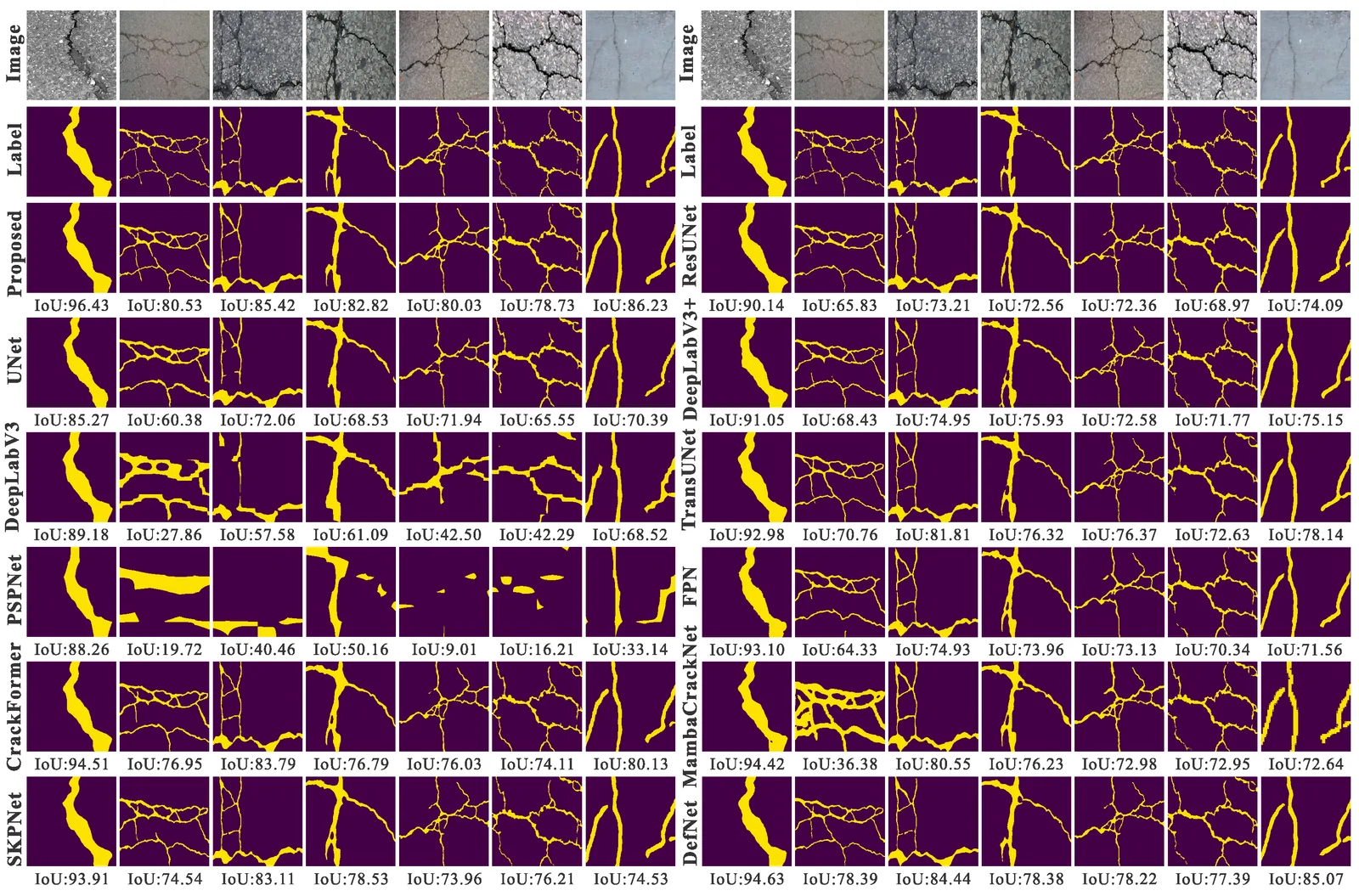
Qualitative segmentation results of the evaluated models on the public datasets.

Table 2 presents the segmentation results on the combined dataset constructed by integrating the four public benchmark datasets. The proposed teacher model achieved the highest performance among all evaluated models, with an mIoU of 0.8087 and an mDice of 0.9028. Its training process also converged stably without significant loss fluctuations, as shown in Figure 9. Among the CNN-based baselines, DeepLabV3+ and FPN achieved the best performance, with mIoU values of 0.7227 and 0.7112, respectively. This performance is attributable to their integration of multi-scale contextual information and high-resolution feature retention. However, their performance on large continuous cracks remained constrained by the limited receptive field inherent to convolutional operations. The Transformer-based models exhibited stronger performance on large-scale cracks through global context modeling. The dual-path design of DefNet (mIoU = 0.7622) further improved feature representation through parallel information processing. The proposed teacher model outperformed DefNet by 6.1% in mIoU. This result indicates that the integration of the VMamba path, the CNN-KAN path and the KAN-based fusion module provides complementary feature representations. The recent KAN-based and Mamba-based competitors are SKPNet and MambaCrackNet. They achieve mIoU values of 0.7606 and 0.7464. These values rank below DefNet and above the remaining CNN-based and Transformer-based baselines. Across the paradigms, CNN-based models capture fine and thin cracks more accurately, while Mamba-based and Transformer-based models segment large and continuous cracks more completely. The proposed dual-path architecture combines these strengths through the CNN-KAN path and the VMamba path, and it exceeds SKPNet by 6.3% and MambaCrackNet by 8.3% in mIoU. As reported in Table 2, the proposed teacher model requires 51% fewer FLOPs than TransUNet. The parameter count of the proposed model is also 34% lower than that of TransUNet. This efficiency is attributable to the linear complexity of the VMamba path. The segmentation results of all models are illustrated in Figure 10.

### 4.2. Model Transfer and Evaluation on Road Crack Datasets

**Figure 11:**
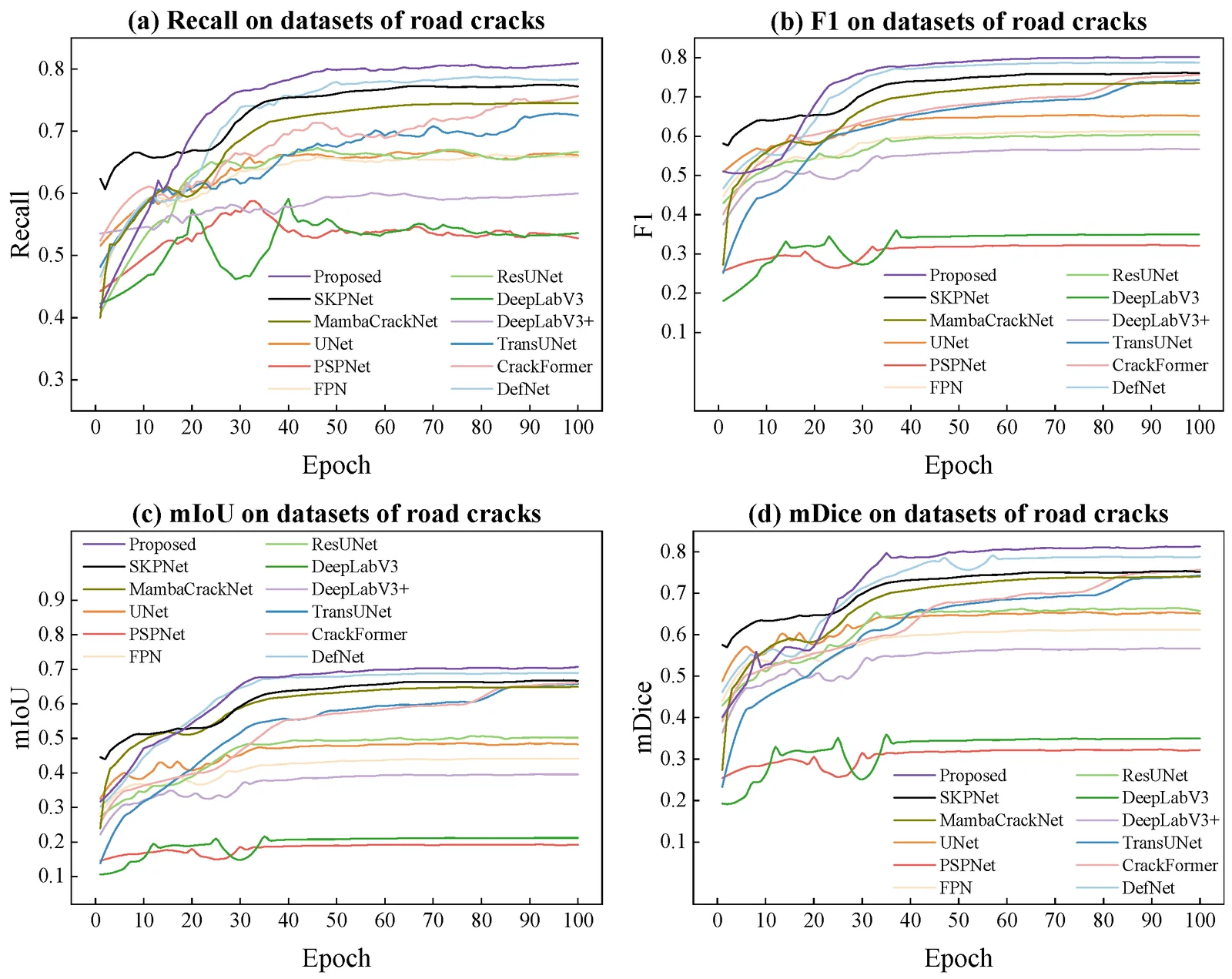
Training curves during transfer learning to the smartphone dataset: (**a**) recall; (**b**) F1; (**c**) mIoU; (**d**) mDice.

Transfer learning was employed to adapt the teacher model to the self-constructed smartphone-captured pavement dataset. This teacher model was pre-trained on the combined public dataset. A progressive unfreezing strategy was adopted. The decoder was fine-tuned first, and the encoder layers were gradually unfrozen thereafter. This strategy preserves the pre-trained feature representations while adapting to the target domain distribution. Because of the severe class imbalance between crack and background pixels, all metrics are computed on the crack class only. The precision, recall, and F1 score are reported together with the crack-class mIoU and mDice. Figure 11 presents the training dynamics of each model during transfer learning.

**Figure 12:**
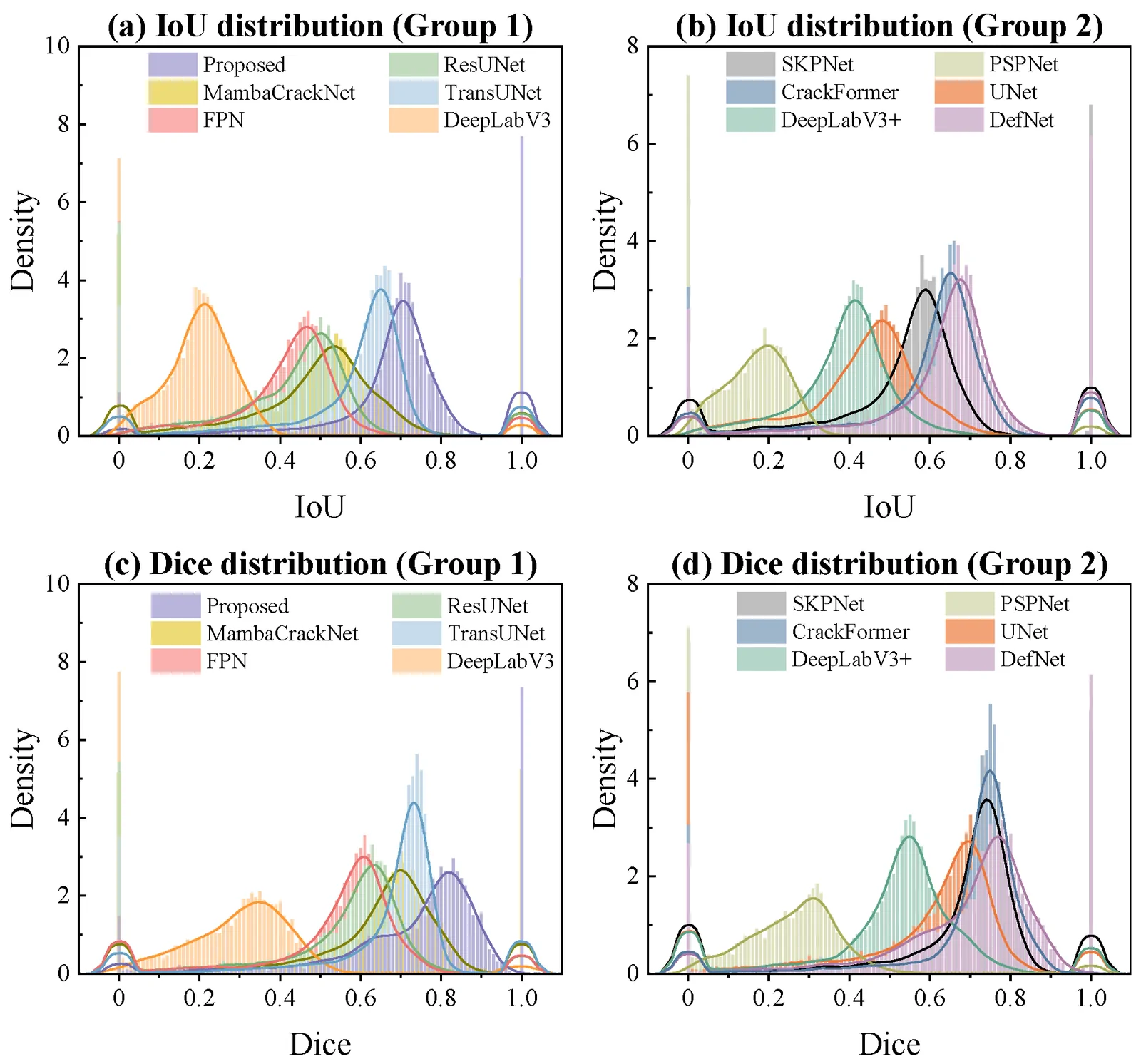
Performance histogram of the evaluated models on the smartphone test subset: (**a**) IoU distribution (Group 1); (**b**) IoU distribution (Group 2); (**c**) Dice distribution (Group 1); (**d**) Dice distribution (Group 2).

As shown in Table 3, all models experience varying degrees of performance degradation after transfer. CNN-based models suffer the most significant degradation. DeepLabV3 drops by 65.4% because its atrous convolution mechanism struggles to extract fine crack features from low-resolution smartphone imagery. Transformer-based models demonstrate stronger domain adaptation through their global self-attention mechanism, which maintains structural crack information under degraded image quality. The recent SKPNet and MambaCrackNet reach mIoU values of 0.6707 and 0.6507 on the smartphone dataset. They remain below the proposed model, which keeps the highest accuracy on both datasets. Figure 12 presents the performance histogram of all segmentation models on the test dataset, further illustrating the performance gap between CNN-based and sequence modeling-based architectures.

**Figure 13:**
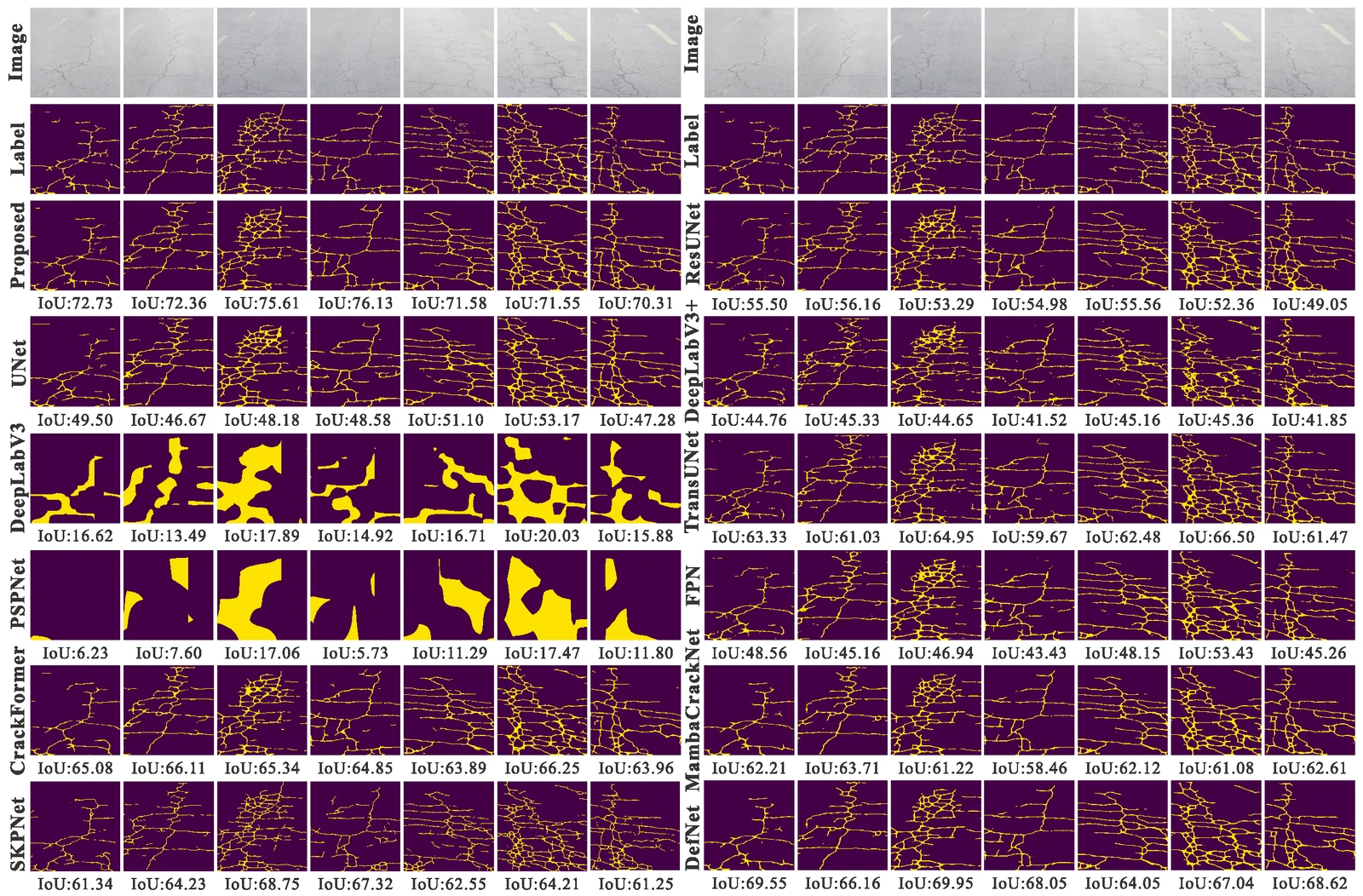
Qualitative segmentation results on the smartphone-captured dataset with ground truth masks.

The proposed teacher model achieves the highest mIoU of 0.7084 among all evaluated models, despite a 12.4% performance decrease relative to its public dataset results. This decrease is attributable to the greater morphological complexity of real-world cracks, which commonly exhibit non-linear mesh-like structures. The teacher model achieves this performance with fewer parameters and FLOPs than TransUNet. This confirms that the VMamba and CNN-KAN paths provide robust domain adaptation capability while maintaining the computational efficiency required for subsequent knowledge distillation. Figure 13 illustrates that the proposed model produces smoother and more semantically consistent crack boundaries compared to baseline models.

### 4.3. Ablation Experiment

**Figure 14:**
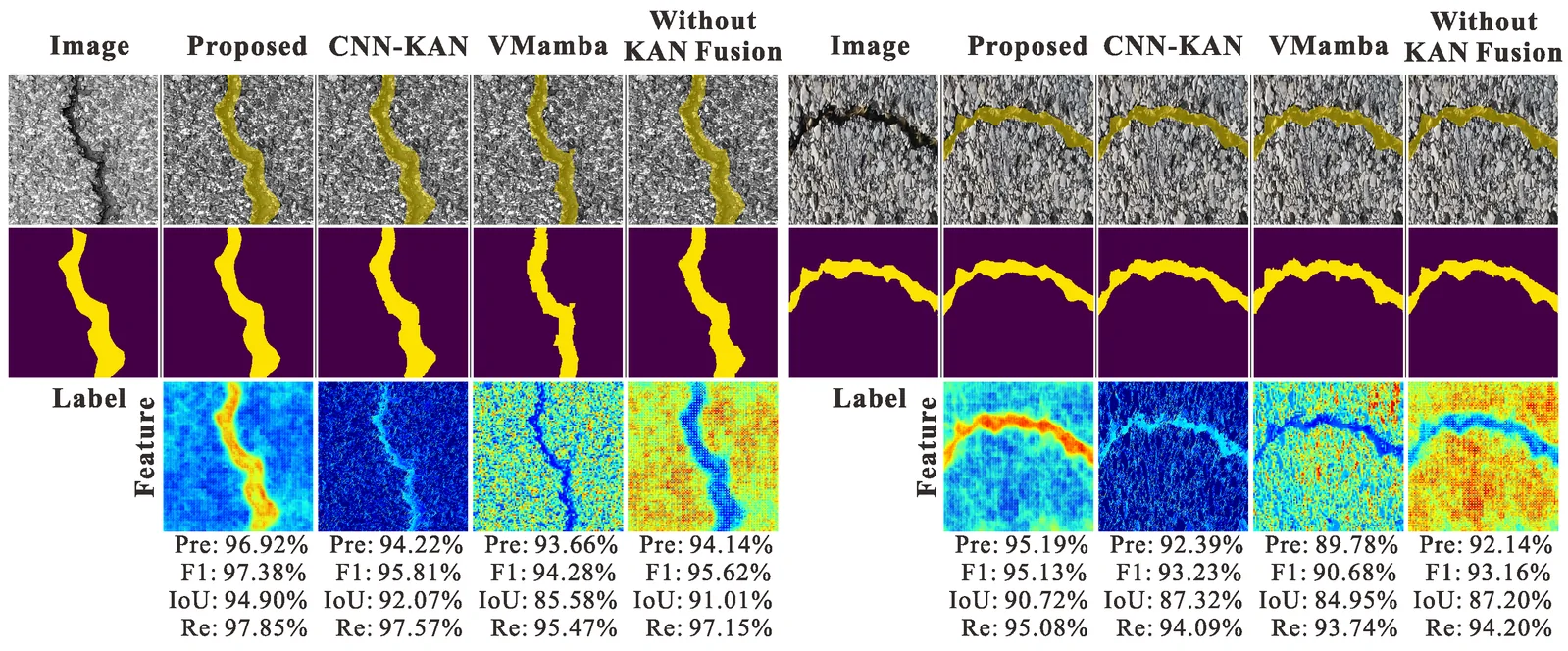
Ablation results of the encoder paths and the KAN fusion module.

Ablation experiments were conducted on both the combined public dataset and the smartphone-captured pavement dataset to verify the contribution of each component to the teacher model. The results are reported in Table 4 and Figure 14. The VMamba encoder alone achieved the lowest performance on both datasets, with mIoU values of 0.6788 and 0.5827, respectively. This result is attributable to its insufficient local receptive field for fine crack detection. Its lower parameter count nevertheless supports its role as an efficient global context modeling component. The CNN-KAN encoder alone achieved mIoU values of 0.7690 and 0.6383. This encoder performed well on fine cracks, but it produced incoherent segmentation masks for large continuous cracks owing to its limited global modeling capacity. Combining the two encoders through simple concatenation, without the KAN-based fusion module, improved the mIoU to 0.7726 and 0.6642. This improvement confirms the complementary nature of the local and global features extracted by the two encoders. The full dual-path architecture with the KAN-based fusion module achieved the highest performance on both datasets, with mIoU values of 0.8087 and 0.7084. This finding shows that the adaptive nonlinear mapping of the fusion module integrates the two heterogeneous encoder representations more effectively than simple concatenation. The consistency of this trend across both datasets further demonstrates the robustness of the proposed fusion strategy under different imaging conditions.

### 4.4. Evaluation of Model Robustness

**Figure 15:**
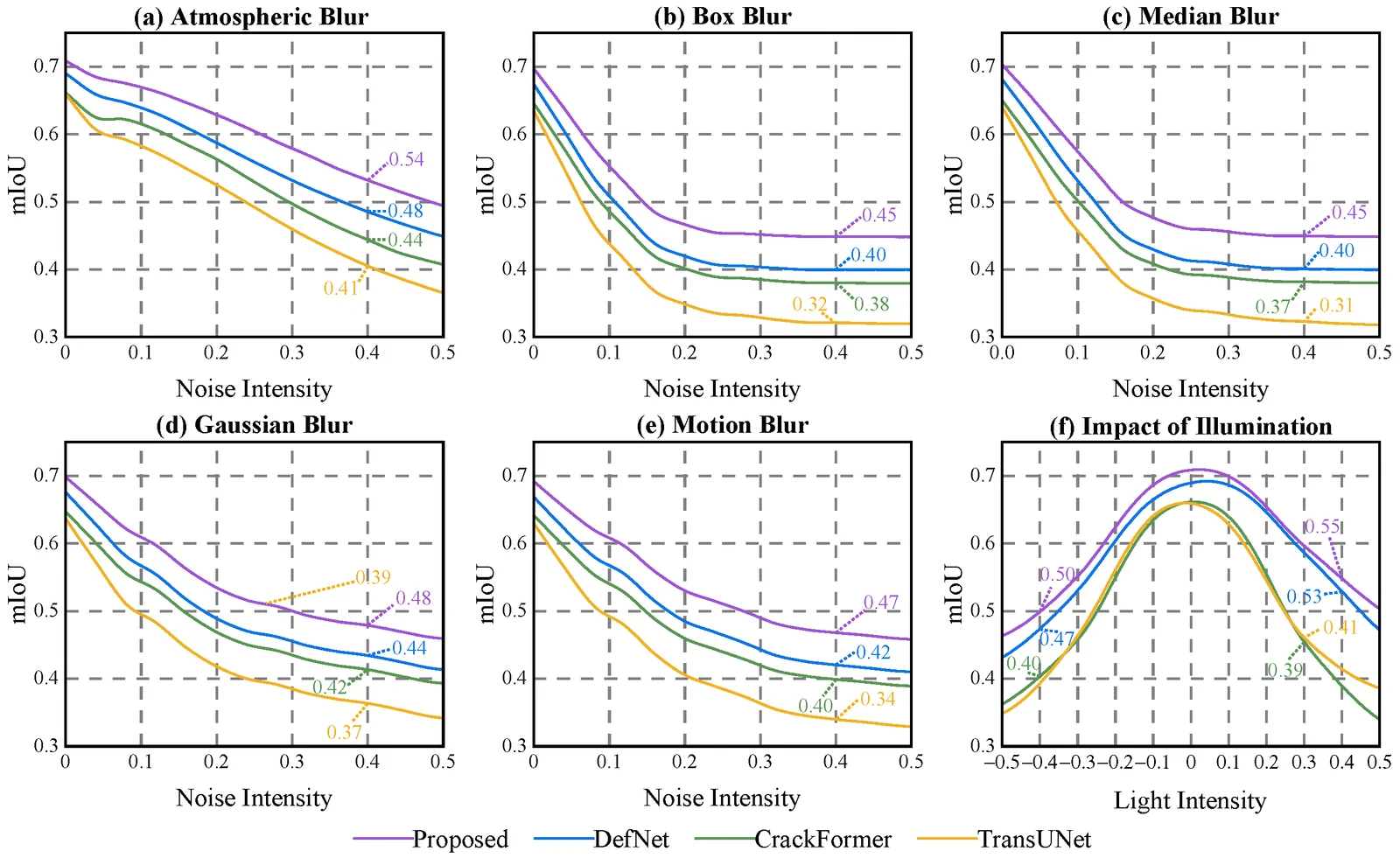
Robustness of the evaluated models under blur and illumination perturbations: (**a**) atmospheric blur; (**b**) box blur; (**c**) median blur; (**d**) Gaussian blur; (**e**) motion blur; (**f**) impact of illumination.

Five perturbation modes representative of real-world smartphone imaging conditions are applied to evaluate the robustness of the teacher model. These modes are atmospheric blur, box blur, median blur, motion blur, and illumination change. The perturbation intensity is normalized by the extreme state of each mode, in which an intensity of 100% corresponds to complete degradation. Results are shown in Figure 15.

Among blur perturbations, atmospheric blur has the least impact. Median blur and box blur cause the sharpest degradation by reducing crack edge sharpness. The proposed dual-path architecture demonstrates stronger blur resistance than single-path Transformer baselines (TransUNet). The CNN-KAN and VMamba paths provide complementary local and global feature extraction. The two paths mutually compensate for feature degradation caused by blur. This robustness advantage cannot be replicated by single-path architectures.

**Figure 16:**
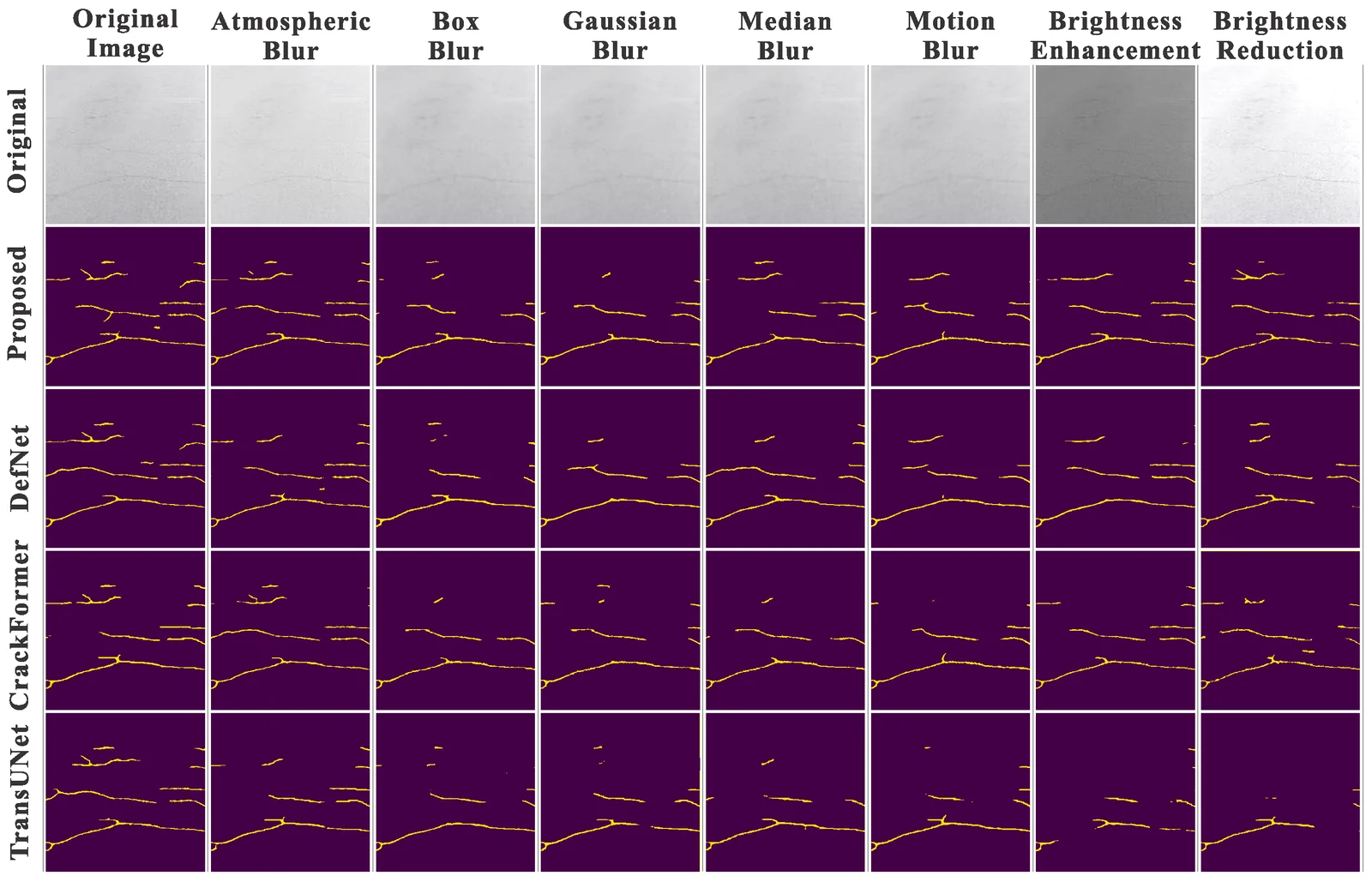
Evaluation of segmentation models under various perturbations.

Under illumination perturbations, the model maintains stable performance across moderate luminance variations. However, it shows greater sensitivity under low-light conditions than under overexposure. This suggests that image acquisition in low-light environments should be avoided. The convolution-augmented Transformer baseline (CrackFormer) shows higher sensitivity to luminance changes due to overfitting on fuzzy crack boundaries. The proposed model exhibits more balanced robustness across illumination conditions. Figure 16 presents the robustness performance of each model at 40% perturbation intensity.

### 4.5. GAN-Driven Optimization of Segmentation Model

**Figure 17:**
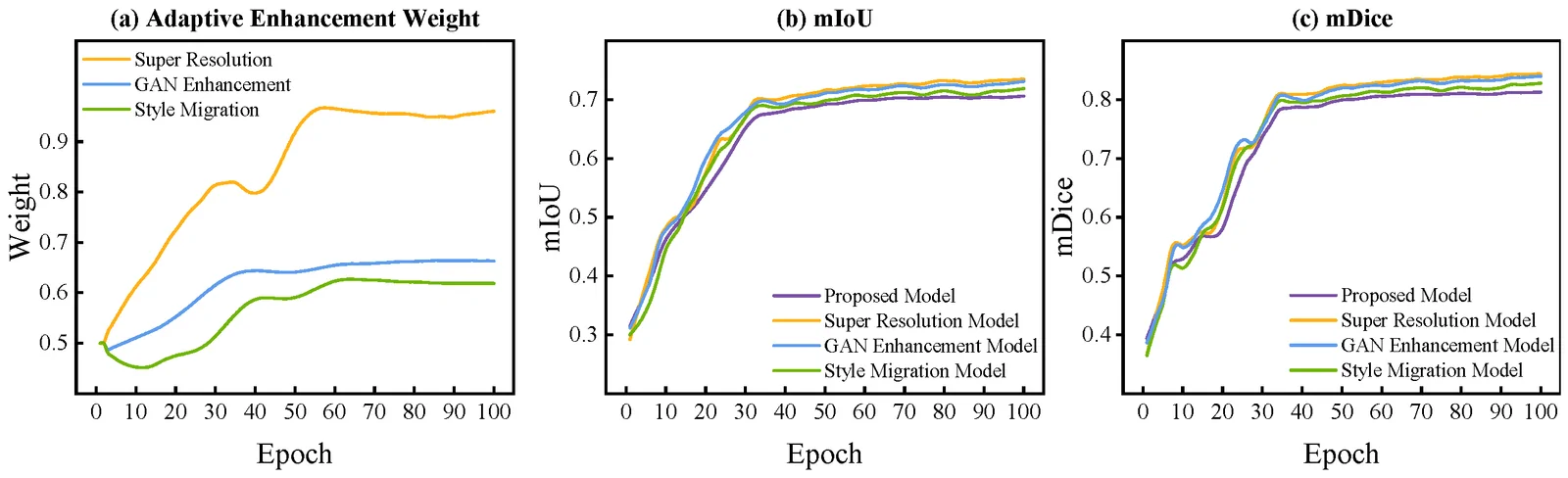
Training curves of the GAN-based enhancement strategies: (**a**) adaptive enhancement weight; (**b**) mIoU; (**c**) mDice.

Three GAN-based enhancement strategies are evaluated on the teacher model to investigate their influence on segmentation performance under real-world smartphone imaging conditions. These strategies are GAN-based image enhancement, CycleGAN style migration, and super-resolution reconstruction. Results are shown in Figure 17.

**Figure 18:**
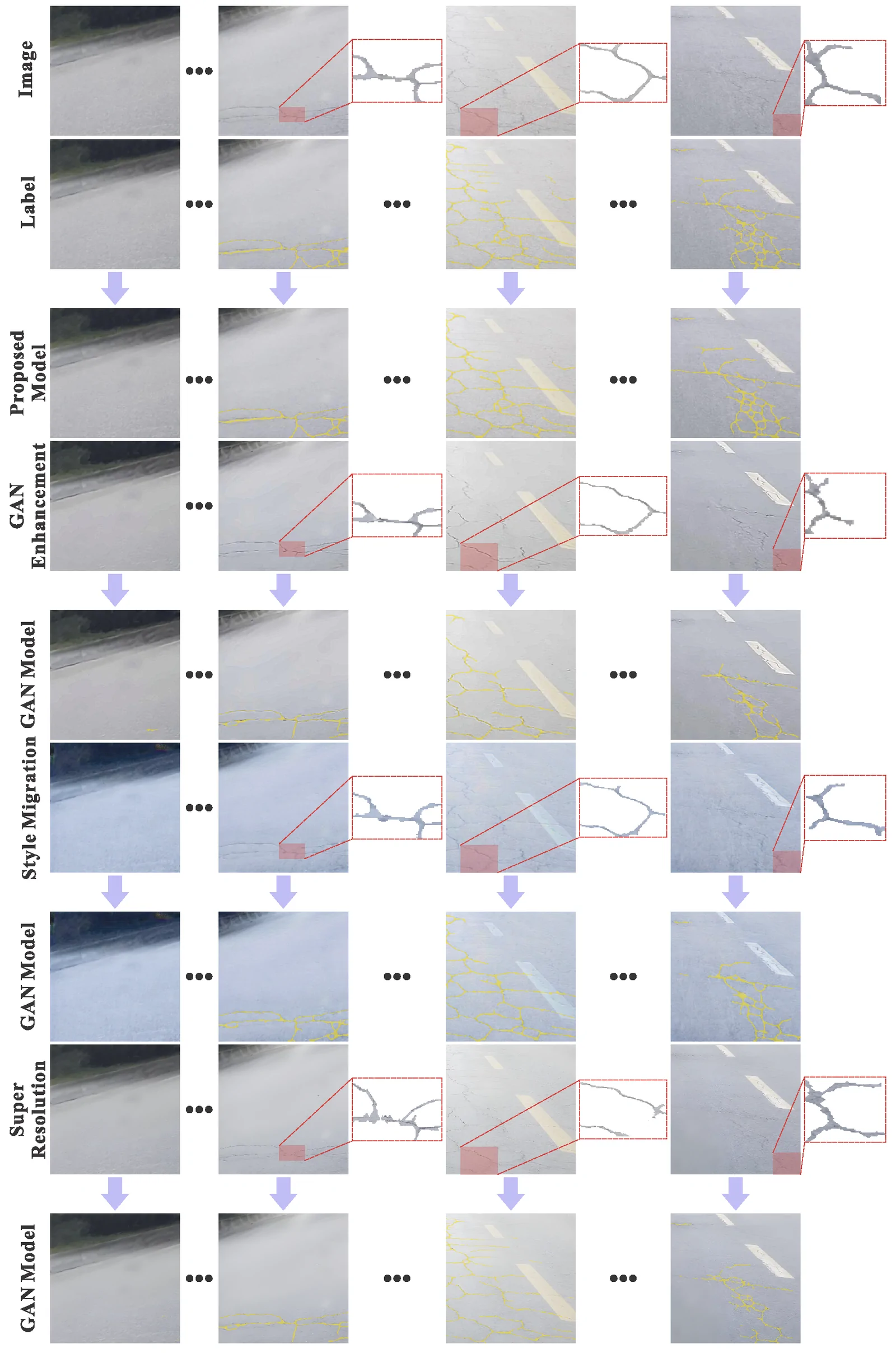
Qualitative evaluation of different GAN models.

As shown in Figure 18, super-resolution reconstruction achieves the greatest improvement, raising mIoU from 0.7084 to 0.7368 (+4.01%) and mDice to 0.8449. This confirms that detail recovery is the most critical factor for crack segmentation in low-resolution smartphone imagery. Style migration (mIoU = 0.7309, +3.18%) and GAN-based image enhancement (mIoU = 0.7195, +1.57%) provide smaller but consistent improvements. Style migration reduces domain shift, while GAN-based enhancement diversifies crack edge representation. GAN enhancement may occasionally cause overfitting to road texture features. Nevertheless, the overall results confirm that all three strategies effectively expand the training distribution and improve the generalization of the teacher model under the challenging imaging conditions of smartphone-captured pavement images.

### 4.6. Deployment of Lightweight Distillation Model

The distillation experiments use the transfer-learned teacher model without GAN-based augmentation, so that the contribution of the distillation framework can be isolated from that of data enhancement. The data enhancement acts at the data level, whereas the distillation acts at the model level. The two components are complementary and can be combined. The ConvLSTM-based knowledge distillation framework compressed the teacher model into five candidate student architectures for smartphone deployment. These architectures included EfficientNet, MobileNet, ShuffleNet, Fast-SCNN and MobileViT. All student models were trained under an identical distillation pipeline and evaluated on the fixed test subset of 265 keyframes defined in Section 2.3.

An ablation study was first conducted with MobileViT as a fixed student backbone to isolate the contribution of each component of the distillation framework. The results are reported in Table 5. When trained without distillation, MobileViT achieved an mIoU of 0.6021. This result reflects the limited representational capacity of the backbone when trained independently on the relatively small smartphone-captured dataset. Applying knowledge distillation without the ConvLSTM module increased the mIoU to 0.6785, indicating that feature-level and response-level knowledge transfer from the teacher model improved student performance. The addition of ConvLSTM-based temporal distillation further increased the mIoU to 0.7078. In both distillation configurations, the teacher model is applied to the same three frames of each clip, so the number of teacher inferences and the amount of soft-label supervision are identical. The configuration without ConvLSTM processes the three frames independently, while the configuration with ConvLSTM aggregates them along the temporal dimension. The gain from 0.6785 to 0.7078 can therefore be attributed to the temporal modeling of the ConvLSTM rather than to additional teacher inferences. Since the backbone architecture remained unchanged across the three configurations, the parameter count and inference time were identical. The observed performance differences can therefore be attributed solely to the distillation strategy. These results indicate that each component of the proposed distillation framework contributes to the final performance.

Table 6 compares the five student architectures under the full distillation pipeline. All student models were trained and evaluated under the identical data partition defined in Section 2.3. This setting allows their accuracy to be compared directly with the baselines in Table 3. MobileViT achieved the closest performance to the teacher model. It retained an mIoU of 0.7078, compared with 0.7084 for the teacher model. MobileViT also reduced the inference time from 99.56 ms to 1.46 ms and reduced the model size from 220.65 MB to 6.01 MB. The teacher model has 57.80 M parameters and an inference time of 99.56 ms. These requirements make it unsuitable for direct mobile deployment. The distillation framework therefore serves as a bridge between the high-accuracy teacher model and the resource-constrained deployment environment. The performance of MobileViT is attributed to two factors. First, MobileViT shares an architectural similarity with the teacher model, as both adopt sequence-based processing. Second, the ConvLSTM-based temporal distillation complements MobileViT’s sequence processing pipeline. EfficientNet exhibited the largest degradation, as its composite scaling strategy is optimized for image classification rather than the elongated morphology of road cracks. Fast-SCNN achieved the fastest inference of 1.02 ms, but it showed notable accuracy loss. MobileNet and ShuffleNet achieved competitive accuracy, but they showed lower efficiency than MobileViT.

**Figure 19:**
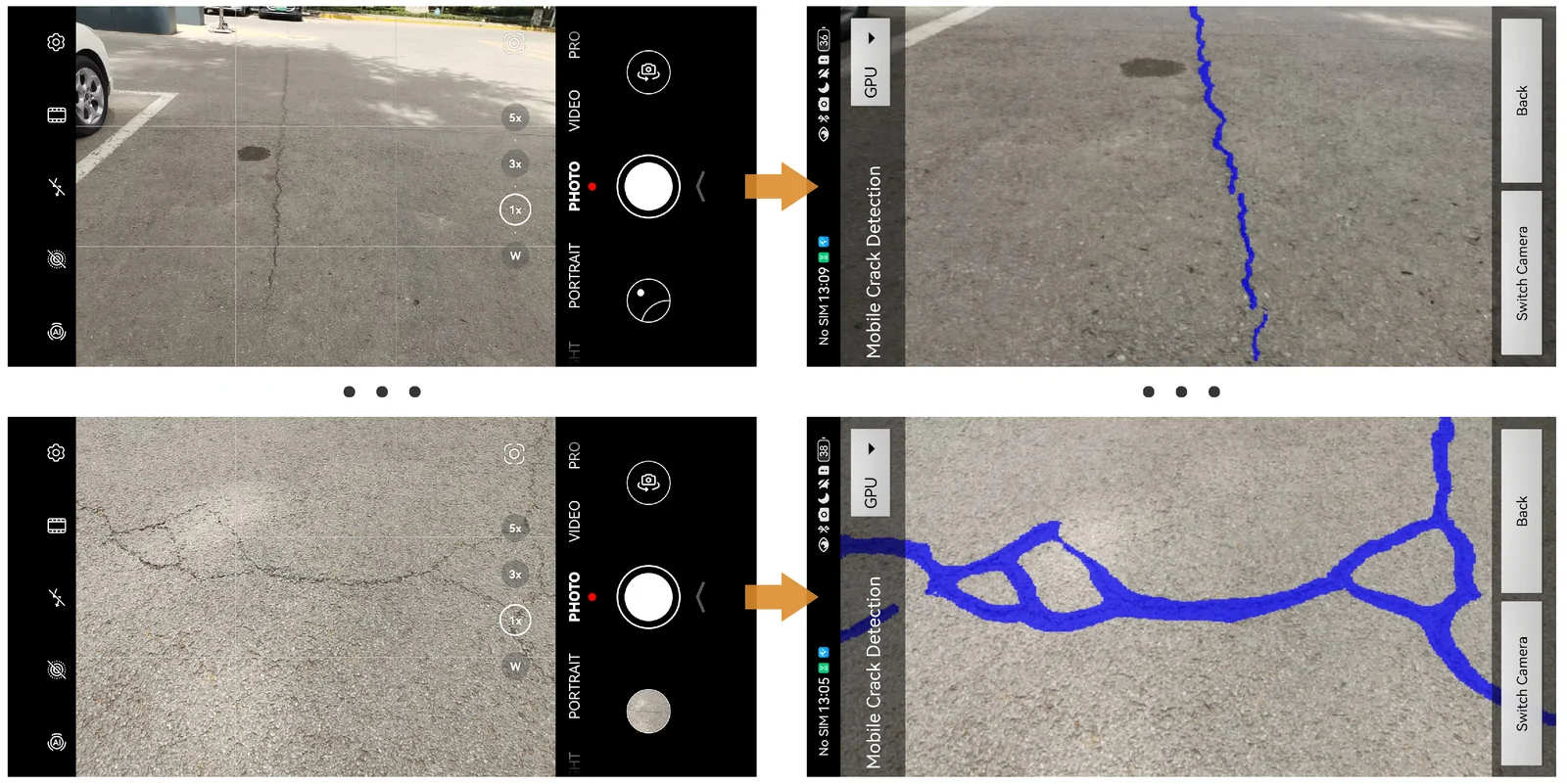
Deployment of the distilled student model on an Android smartphone.

The comparison between Table 3 and Table 6 indicates that three distilled student models have higher mIoU values than the strongest baseline model DefNet (mIoU = 0.6899). These student models are MobileViT (mIoU = 0.7078), ShuffleNet (mIoU = 0.6975) and MobileNet (mIoU = 0.6954). This result is notable because the student models have far fewer parameters than the baseline model. This outcome occurs because the knowledge distillation framework transfers the domain-adaptive representation learned by the teacher model on smartphone-captured crack images. This transfer enables lightweight student models to achieve higher accuracy than the heavier baseline models adapted through transfer learning under the identical partition, although the two groups differ in architecture as well as in training strategy. As shown in Figure 19, the distilled MobileViT student model was subsequently deployed on an Android smartphone using the ncnn framework. The inference times of 99.56 ms and 1.46 ms in Table 6 are measured on the RTX 4090 GPU, and they represent the teacher latency and the student latency on the GPU rather than the on-device latency. On the smartphone, the deployed student model achieves 15 to 20 frames per second, which corresponds to about 50 to 67 ms per frame. The on-device latency is higher than the GPU latency, because the smartphone has far less compute than the RTX 4090. This on-device speed still meets the real-time requirement for on-site crack detection and the student model maintains an mIoU of 0.7078.

## 5. Conclusions

This study proposes a two-stage framework for real-time road crack detection on smartphones. The framework combines a dual-path teacher model with a knowledge-distilled lightweight student model. The main conclusions are summarized as follows.

(1) A teacher model is constructed by integrating a CNN-KAN path and a VMamba path through a dedicated KAN-based fusion module. The teacher model achieves an mIoU of 0.8087 and an mDice of 0.9028 on the combined public datasets. Through transfer learning, it achieves an mIoU of 0.7084 on the smartphone-captured pavement dataset, outperforming all eleven baseline models including DefNet. Ablation experiments confirm that the KAN-based fusion module integrates the two heterogeneous feature streams more effectively than simple concatenation.

(2) The teacher model demonstrates strong robustness across blur and illumination perturbations representative of real-world smartphone imaging conditions. The dual-path design provides mutual feature compensation under degraded image quality. Among the GAN-based augmentation strategies, super-resolution reconstruction achieves the greatest improvement of 4.01% in mIoU by recovering high-frequency crack details lost in low-resolution images.

(3) A ConvLSTM-based knowledge distillation framework compresses the teacher model into a mobile-deployable MobileViT student model. This compression reduces the parameter count from 57.80 M to 1.57 M, reduces the model size from 220.65 MB to 6.01 MB, and reduces the inference time from 99.56 ms to 1.46 ms on the same GPU, while retaining an mIoU of 0.7078. On-device deployment through the ncnn framework achieves 15 to 20 frames per second on an Android smartphone. Under the identical data partition, the distilled student model surpasses the strongest baseline DefNet. This outcome shows that distillation from a domain-adapted teacher is more effective than training from scratch for lightweight models on limited field data. An ablation study confirms that the ConvLSTM-based temporal distillation, which aggregates crack features across successive frames, provides a measurable contribution beyond feature-level and response-level transfer.

## Figures and Tables

**Table 1:** Per-dataset segmentation results on the four public benchmark datasets.

Dataset	Crack500		DeepCrack		CFD		CrackTree	
**Metric**	**mIoU**	**mDice**	**mIoU**	**mDice**	**mIoU**	**mDice**	**mIoU**	**mDice**
Proposed	0.82	0.89	0.86	0.92	0.76	0.86	0.72	0.82
SKPNet	0.75	0.86	0.80	0.89	0.72	0.83	0.70	0.83
MambaCrackNet	0.73	0.85	0.77	0.87	0.68	0.81	0.67	0.79
UNet	0.66	0.77	0.68	0.83	0.63	0.79	0.57	0.70
ResUNet	0.69	0.80	0.71	0.85	0.65	0.78	0.61	0.74
PSPNet	0.47	0.63	0.53	0.68	0.40	0.58	0.34	0.52
FPN	0.67	0.81	0.67	0.85	0.66	0.80	0.63	0.75
DeepLabV3	0.62	0.72	0.61	0.75	0.56	0.68	0.50	0.64
DeepLabV3+	0.65	0.83	0.68	0.86	0.67	0.81	0.62	0.76
TransUNet	0.70	0.82	0.71	0.84	0.69	0.77	0.63	0.79
CrackFormer	0.72	0.80	0.76	0.82	0.68	0.81	0.73	0.81
DefNet	0.76	0.84	0.79	0.88	0.71	0.82	0.70	0.83

**Table 2:** Evaluation of semantic segmentation models on public datasets.

Model	Params (M)	FLOPs (G)	P (%)	R (%)	$F_{1}$ (%)	mIoU	mDice
Proposed	57.80	60.07	90.29	88.99	90.10	0.8087	0.9028
SKPNet	31.14	168.02	86.82	85.99	86.40	0.7606	0.8640
MambaCrackNet	41.78	219.50	81.84	89.46	85.48	0.7464	0.8548
UNet	31.04	100.27	80.19	80.70	79.77	0.6634	0.7977
ResUNet	57.57	66.72	81.85	83.20	81.96	0.6944	0.8196
PSPNet	19.99	34.57	64.17	70.98	64.34	0.4742	0.6434
FPN	16.07	55.81	85.12	82.37	83.13	0.7112	0.8313
DeepLabV3	13.76	22.95	71.24	83.26	76.12	0.6145	0.7312
DeepLabV3+	14.46	52.06	86.67	82.44	83.90	0.7227	0.8390
TransUNet	87.91	122.47	84.72	83.18	81.77	0.6989	0.8177
CrackFormer	56.60	81.54	87.21	83.81	83.95	0.7324	0.8395
DefNet	60.75	71.26	87.49	85.64	86.51	0.7622	0.8651

**Table 3:** Evaluation of segmentation models on the smartphone-captured road crack dataset.

Model	P (%)	R (%)	$F_{1}$ (%)	mIoU ^∗^ (μ, σ)	mDice ^∗^ (μ, σ)
Proposed	81.71	82.90	80.41	0.7084, 0.2989	0.8141, 0.2648
SKPNet	75.21	77.86	76.32	0.6707, 0.3004	0.7555, 0.2719
MambaCrackNet	72.45	74.53	73.71	0.6507, 0.3016	0.7418, 0.2628
UNet	61.06	69.34	65.61	0.4883, 0.3307	0.6561, 0.3039
ResUNet	57.16	69.62	60.73	0.5334, 0.3391	0.6673, 0.3051
PSPNet	31.55	67.58	32.47	0.1938, 0.3279	0.3247, 0.4308
FPN	57.98	67.34	61.35	0.4425, 0.3367	0.6135, 0.3026
DeepLabV3	28.67	64.63	35.04	0.2124, 0.2759	0.3504, 0.2496
DeepLabV3+	51.36	65.88	56.85	0.3972, 0.3440	0.5685, 0.3080
TransUNet	76.58	75.01	74.52	0.6591, 0.2993	0.7452, 0.2712
CrackFormer	76.55	77.41	75.77	0.6614, 0.2790	0.7577, 0.2548
DefNet	78.01	79.52	78.88	0.6899, 0.3140	0.7888, 0.2779

^∗^ The reported IoU and Dice are computed on the crack class only.

**Table 4:** Evaluation of feature fusion and encoder effectiveness.

Dataset			Public Datasets		Road Crack Datasets	
**CNN-KAN**	**VMamba**	**KAN Fusion**	**mIoU**	**mDice**	**mIoU**	**mDice**
✓	✓	✓	0.8087	0.9028	0.7084	0.8141
✓	✗	✗	0.7690	0.8733	0.6383	0.7523
✗	✓	✗	0.6788	0.7927	0.5827	0.7141
✓	✓	✗	0.7726	0.8765	0.6642	0.7765

✓ indicates that the component is included; ✗ indicates that it is excluded.

**Table 5:** Ablation study of the knowledge distillation framework.

Training Strategy	ConvLSTM	Params (M)	Inference Time (ms)	mIoU	mDice
Without distillation	✗	1.57	1.46	0.6021	0.7092
With distillation	✗	1.57	1.46	0.6785	0.7867
With distillation	✓	1.57	1.46	0.7078	0.8137

✓ indicates that the ConvLSTM module is used; ✗ indicates that it is not.

**Table 6:** Evaluation of performance in lightweight segmentation models.

Model	Params (M)	Size (MB)	Inference Time ^∗^ (ms)	mIoU	mDice
Proposed	57.80	220.65	99.56	0.7084	0.8141
EfficientNet	2.44	9.33	1.60	0.5561	0.7059
MobileNet	3.23	12.38	6.64	0.6954	0.8056
ShuffleNet	4.41	16.87	7.18	0.6975	0.8070
Fast-SCNN	4.68	18.98	1.02	0.6530	0.7770
MobileViT	1.57	6.01	1.46	0.7078	0.8137

^∗^ The inference time is measured on the RTX 4090 GPU.

## Data Availability

The source code of this study is publicly available on GitHub at https://github.com/MengzhaoNie/Smartphone-Crack-Detection (accessed on 6 August 2026). The self-collected smartphone pavement dataset is publicly available on Figshare at https://doi.org/10.6084/m9.figshare.33090545 (accessed on 6 August 2026) under a CC BY 4.0 license. The four public benchmark datasets used in this study are available from their original sources, which are cited in the references.
